# A network pharmacology-based approach and molecular docking study to explore the therapeutic potential of a nutraceutical formula (Vernolac) in the treatment of cancer

**DOI:** 10.1371/journal.pone.0352420

**Published:** 2026-07-01

**Authors:** Sandani De Vass Gunawardane, Matheen Muhammadh Milhan, Poorni Chanuka Rathnayake, Prabudhi S. Garusinghe, Kavishka S. Gunaratne, Anusha Kanagasundaram, T. M. D. Darshanamala, Duvinika Chalani Senevirathne, Shalini Kaushalya Wijerathne, R. P. C. D. Perera, Kanishka Sithira Senathilake, Umapriyatharshini Rajagopalan, Kamani Hemamala Tennakoon, Sameera Ranganath Samarakoon

**Affiliations:** Institute of Biochemistry, Molecular Biology and Biotechnology, University of Colombo, Colombo, Sri Lanka; Lawrence Livermore National Laboratory, UNITED STATES OF AMERICA

## Abstract

Vernolac is a commercially available polyherbal nutraceutical capsule containing *Vernonia zeylanica* aerial parts, *Nigella sativa* seeds, *Hemidesmus indicus* roots, *Leucas zeylanica* aerial parts, and *Smilax glabra* rhizome. Herbal formulations, organic extracts, and isolated phytochemicals from these plants have demonstrated anticancer properties. However, the mechanisms underlying Vernolac’s anticancer activity as a polyherbal formulation remain largely unexplored. This study utilized an integrative network pharmacology-based approach, supported by *in vitro* experiments, to investigate Vernolac’s anticancer potential. Phytochemicals were retrieved from databases, screened for drug-likeness and oral bioavailability using SwissADME, yielding 155 drug-like compounds, and their protein targets were predicted using SwissTargetPrediction. The intersection of phytochemical targets with cancer-related targets from GeneCards identified 137 common targets. Protein-protein interaction analysis using STRING and Cytoscape revealed fourteen key hub nodes, including AKT1, BCL2, CASP3, CTNNB1, EGFR, ESR1, GAPDH, HSP90AA1, HSP90AB1, IL6, JUN, SRC, STAT3, and TNF. Network analyses highlighted key phytochemicals, including vernolactone, thymoquinone, quercetin, nigellidine, α-hederin, and carvacrol. GC–MS profiling of the supercritical CO_2_ extract of Vernolac revealed a diverse phytochemical composition enriched with terpenes, fatty acids, and sterols, including the key constituents stigmasterol, thymoquinone, and carvacrol. Gene Ontology (GO) and Kyoto Encyclopedia of Genes and Genomes (KEGG) enrichment analyses revealed significant enrichment of the identified targets across multiple cancer pathways. Molecular docking and dynamics simulations identified novel target-ligand interactions, such as vernolactone-β-catenin and α-hederin-CDK4. The Sulforhodamine B assay demonstrated selective antiproliferative activity of Vernolac extract against cancerous cell lines MCF-7 (IC_50_ = 54.01 ± 0.02 μg/mL), Caco-2 (IC_50_ = 85.52 ± 0.13 μg/mL), NTERA-2 cl.D1 (IC_50_ = 42.41 ± 0.06 μg/mL), and non-cancerous MCF-10A (IC_50_ = 803.5 ± 0.03 μg/mL). Collectively, network analysis suggests that phytochemicals in Vernolac may exert anticancer effects through multiple cancer-related pathways, including those associated with apoptosis, immune modulation, oxidative stress, inflammation, and cell proliferation. Furthermore, the identified targets and enriched pathways suggest a potential role in modulating drug resistance and treatment response, providing a computational basis for its application as an adjunct to conventional cancer therapies and warranting further investigation in preclinical and clinical settings.

## Introduction

Cancer remains one of the leading causes of death worldwide. Despite significant development in early detection and treatment strategies, the global cancer burden continues to escalate [1,2]. While conventional treatments like surgery, chemotherapy, radiation, and targeted therapies remain standard approaches, they are frequently associated with unpleasant side effects. [3]. Furthermore, the emergence of therapy resistance further complicates treatment outcomes, leading to treatment failure [4]. These challenges underscore the urgent need for novel therapeutic strategies that are both effective and well-tolerated.

Natural products have gained significant attention in cancer therapy due to their broad-spectrum therapeutic possibilities [5]. Many natural products derived from plants, marine organisms, fungi, and microbes exhibit anticancer effects by modulating key cellular processes, including apoptosis, proliferation, angiogenesis, and immune regulation [6]. In this context, nutraceuticals have emerged as promising adjuncts to conventional cancer therapies [7]. These products are particularly attractive for their potential to exert multi-targeted effects while protecting healthy tissues, making them promising candidates for long-term use in cancer management [8].

Network pharmacology is an emerging discipline in pharmacological research, offering a powerful platform to decode the complex interactions between compounds, targets, and disease-related pathways [9]. Unlike the traditional “one drug-one target” approach, network pharmacology embraces the multi-component, multi-target, and multi-pathway nature [9,10]. Network pharmacology has been increasingly applied to traditional polyherbal formulations, particularly in Indian Ayurvedic [11,12] and Traditional Chinese Medicine (TCM) [9,10], to explore their complex therapeutic mechanisms. Previous studies have highlighted the value of network pharmacology as a modern systems-level approach to rationalizing traditional formulations and guiding mechanistic discovery [11,13–15].

Sri Lankan traditional medicine similarly embodies a long-standing practice of combining herbs and polyherbal decoctions for the treatment of various diseases. In this context, Vernolac represents a contemporary formulation that emerges from this ethnomedical lineage. It is a polyherbal nutraceutical capsule of Fadna Life Sciences (Pvt). Ltd (106/6B, Araliya Uyana, Depanama, Pannipitiya, Sri Lanka), currently available in the Sri Lankan market for the management of cancer. The formulation comprised five medicinal plants with ethnomedical relevance, namely *Vernonia zeylanica* aerial parts, *Nigella sativa* seeds, *Smilax glabra* rhizome, *Leucas zeylanica* aerial parts, and *Hemidesmus indicus* roots [16]. Notably, a polyherbal decoction comprising *N. sativa* seeds, *H. indicus* roots, and *S. glabra* rhizomes, used in equal proportions, has been employed for generations by a family of Ayurvedic physicians in Sri Lanka for the management of cancers, underscoring its historical ethnopharmacological relevance [17]. Vernolac incorporates these same three medicinal plants along with two other plants, as mentioned above [16].

Although the anticancer potential of most of the phytochemicals, several herbal formulations, and organic extracts of the aforementioned plants has been reported in previous studies [17–25], the anticancer mechanisms of Vernolac, as a polyherbal formulation, remain unexplored.

**Fig 1:**
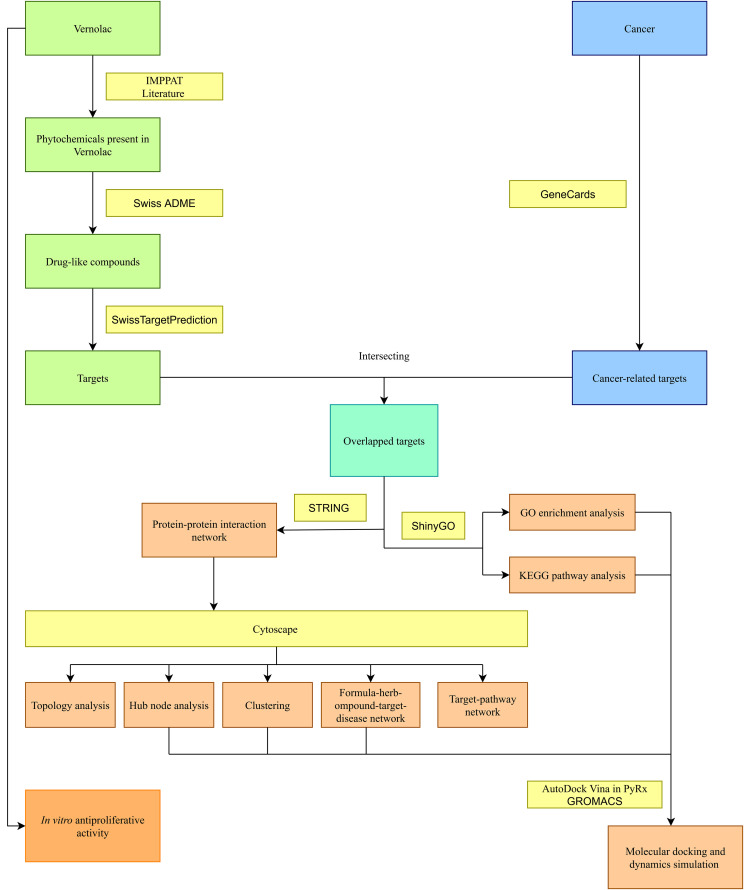
Workflow for investigation of the anticancer mechanisms of action of Vernolac in the treatment of cancer.

Given the success of network pharmacology in unravelling the mechanistic basis of traditional herbal formulations in Chinese and Indian traditional medicinal systems, applying this approach to Vernolac provides a rational and systematic pathway to decode its multi-component, multi-target, and multi-pathway therapeutic potential in the treatment of cancer. Vernolac, therefore, represents a timely and relevant candidate for such an investigation, particularly as this study constitutes the first network pharmacology-based investigation of a Sri Lankan polyherbal formulation. Accordingly, in the present study, a systematic network pharmacology-based approach was employed to investigate the anticancer mechanisms of action of Vernolac. By integrating target prediction, protein-protein interaction analysis, and pathway analysis, this study aimed to explore the key targets and pathways modulated by Vernolac. Moreover, molecular docking and dynamics simulation studies were employed to further validate interactions between phytochemicals in Vernolac and key cancer targets. Additionally, the cytotoxicity of Vernolac was evaluated in both cancer cells and normal cells. The workflow of the network pharmacology approach is shown in Fig 1.

## Materials and methods

### Acquisition of phytochemicals in Vernolac

Vernolac is composed of *N. sativa*, *S. glabra*, *L. zeylanica*, *V. zeylanica*, and *H. indicus* [16,25]. Compounds of *N. sativa*, *S. glabra,* and *H. indicus* were retrieved from the Indian Medicinal Plants, Phytochemistry and Therapeutics 2.0 (IMPPAT 2.0) database (https://cb.imsc.res.in/imppat/). Literature mining also supplemented the information from the database. Since there is no information on *L. zeylanica* and *V. zeylanica* in IMPPAT 2.0, their compounds were retrieved via a literature search. After removing duplicates, the chemical structure and canonical SMILES of each compound were obtained from the PubChem database (https://pubchem.ncbi.nlm.nih.gov/). The chemical structures of compounds without information in the PubChem database were drawn using MarvinSketch from ChemAxon (https://chemaxon.com/marvin).

### Prediction of drug-likeness of Vernolac compounds

Despite remarkable *in vitro* results, some phytochemicals have been shown to have minimal or negligible *in vivo* activity, leading to poor absorption and, consequently, low bioavailability [26]. Drug discovery widely uses absorption, distribution, metabolism, and excretion (ADME) studies to optimize the balance of properties required to convert leads into effective medications [27]. Therefore, ADME characteristics of phytochemicals must be considered. In the present study, drug likeness and bioavailability of compounds were predicted using SwissADME, a free web tool to evaluate pharmacokinetics, drug-likeness, and medicinal chemistry friendliness of small molecules.

The chemical structures or canonical SMILES of each compound were imported into SwissADME (http://www.swissadme.ch/) to obtain drug-likeness status and bioavailability score. The active compounds were filtered by the criteria of the Lipinski rules for drug-likeness status: ‘Yes’ and the oral bioavailability score of ≥ 0.30. Compounds that align with the above criteria were selected for target prediction.

### Target prediction of compounds and cancer

The structure or SMILES of each phytochemical was imported into SwissTargetPrediction (http://www.swisstargetprediction.ch/), a free web tool that predicts the most probable protein targets of small molecules. The species was set to “*Homo sapiens*,” and the target data for each compound were exported in CSV format. Targets with a probability greater than zero were then extracted for further analysis. The list of targets was imported into the UniProt database (https://www.uniprot.org/) to ensure standardized, consistent protein names. Cancer-related genes were obtained by searching the GeneCards database (https://www.genecards.org/) with the keyword ‘Cancer’.

### Acquisition of common targets

The Vernolac and cancer-related targets were uploaded to the Venny 2.1.0 online tool (https://bioinfogp.cnb.csic.es/tools/venny/) to identify targets overlapping between Vernolac and cancer.

### Selection of core targets for further analyses

Given the high number of common targets, a stepwise filtering strategy was implemented to identify core targets within the large protein-protein interaction (PPI) network. This approach utilized network topology parameters, with a focus on degree centrality, to prioritize highly connected and biologically significant nodes.

### Construction of the preliminary protein-protein interaction (PPI) network

The UniProt IDs of common targets of cancer and Vernolac were uploaded into the STRING database (https://string-db.org/, version 12.0) for initial PPI network construction, selecting the ‘multiple protein’ option on the database, and the species was selected as “*Homo sapiens*”. The confidence score was set to 0.9 to ensure high-confidence interactions. This threshold yielded a network consisting of 679 nodes (proteins) and 4934 edges (interactions).

### Network analysis and filtering

Cytoscape is an open-source software platform for analyzing and visualizing complex networks (https://cytoscape.org/). Cytoscape version 3.10.3, along with its various plugins, was utilized in this study.

The STRING PPI network was imported into Cytoscape for the topology analysis. To identify core targets, the network topology was analyzed using the CytoNCA plugin (https://apps.cytoscape.org/apps/cytonca), a Cytoscape plugin integrating calculation, evaluation and visualization analysis for multiple centrality measures [28]. Weighted analysis was performed by incorporating interaction scores from the STRING database as edge weights to reflect the reliability and strength of protein-protein interactions.

Degree centrality (DC), a measure of the number of direct connections (edges) a node has with other nodes in the network, was used to identify core targets within the network. The DC highlights hub proteins that are likely to be biologically significant due to their extensive connectivity within the PPI network, often participating in multiple pathways and regulating essential biological processes.

In the present study, nodes with a DC ≥ 20 were selected to ensure the retention of core proteins with relatively high connectivity within the network. This cutoff was selected based on the distribution of DC values, where a threshold representing the top 20% of nodes in the network was applied, with DC ≥ 20 defining the core targets in the network. This approach enabled a balance between retaining biologically important targets and simplifying the network by excluding low-connectivity nodes. After filtering, the network was reduced to 137 nodes representing the core targets with high connectivity. The downstream analyses were conducted on the refined network that contains 137 nodes and their interactions.

### Construction and network analyses of the core target PPI network

The core target PPI network was constructed using the STRING database. The core target PPI network was imported into Cytoscape, and network parameters were analyzed. Topology parameters, including betweenness centrality (BC), closeness centrality (CC), and degree centrality (DC), were analyzed with weight using the CytoNCA plugin.

### Identification of hub nodes

The CytoHubba plugin (https://apps.cytoscape.org/apps/cytohubba) was used to identify top hub proteins. In this study, five different methods of the CytoHubba plugin were employed, including two global rank methods (betweenness and closeness) and three local rank methods (degree, maximal clique centrality (MCC), and maximum neighborhood component (MNC)), as described in a previous study [29]. While global rank methods consider the relationship between each node and the entire network, local rank methods calculate the score of a node within a network based on the proximity of the node to its direct neighbors [30]. The top 20 hub nodes were obtained using each method, and the common hub proteins were identified by combining the five CytoHubba methods.

### Identification of clusters of the PPI network

In complex PPI networks, some proteins are densely connected, forming clusters. Proteins in a cluster have the same or similar functions and frequently interact with each other, creating a functional module within the network. The clustering of the PPI network was performed using the Molecular Complex Detection (MCODE) Cytoscape plugin (https://apps.cytoscape.org/apps/mcode). MCODE is an algorithm that effectively finds densely connected groups within a molecular interaction network, based on interaction data.

The PPI network was imported to Cytoscape for clustering using the MCODE. Parameters, including degree cutoff (2), node score cutoff (0.2), k-core cutoff (2), and max depth (100), were adjusted, and the haircut filter was enabled. The clusters were visualized, and the topology parameters of each cluster were calculated using MCODE and the Analyze Network Cytoscape plugin.

### Construction and topology analysis of Formula-Herb-Compound-Target-Disease network

The formula-herb-compound-target-disease network was constructed using Cytoscape 3.10.3 software to visualize the complex interactions between compounds and cancer-related protein targets. Separate data files were prepared for the formula-herb, herb-compound, compound-target, target-target, and target-disease relationships using Microsoft Excel. The data files were then imported into Cytoscape to merge networks, constructing the interaction network of Vernolac in cancer treatment. In this graphical network, the formula, herbs, compounds, targets, and disease were denoted as nodes, and the edges represent formula-herb-compound-target-disease interactions. The Analyze Network plug-in in Cytoscape was used to assess the topology parameters (degree) of the network.

### GO and KEGG pathway enrichment analysis

Enrichment analysis on the core target proteins was carried out using the ShinyGO (version 0.81) web-based application (https://bioinformatics.sdstate.edu/go/). The ShinyGO is a large annotation and pathway database for Gene Ontology (GO) functional enrichment analysis and Kyoto Encyclopedia of Genes and Genomes (KEGG) pathway enrichment analysis [31].

The three major GO terms analyzed were the biological processes (BP), cellular components (CC), and molecular functions (MF). KEGG pathway enrichment analysis was used to identify molecular biological pathways enriched by the core targets of Vernolac and cancer. The false discovery rate (FDR) cutoff was set to 0.05, and the top 20 BP, CC, and MF GO terms and the top 20 KEGG pathways were selected based on the fold enrichment. Bubble charts with fold enrichment, FDR, and gene counts were used for the graphical representation of the top 20 GO terms and KEGG pathways.

### Construction of the target-pathway network

To effectively illustrate the relationship between protein targets and the highly enriched pathways in which they are involved, the top 20 KEGG pathways were selected to construct a target-pathway network. Target-target and target-pathway data sheets were prepared in Microsoft Excel. Then, Cytoscape was used to merge the imported files and generate a comprehensive target-pathway network that highlights key biological connections.

### Molecular docking

Based on the results of network analysis, pathway enrichment, and hub nodes analysis, ten high-priority targets central to cancer-associated signaling pathways were selected for molecular docking and dynamics simulations. Accordingly, X-ray crystal structures for AKT1 (PDB ID: 3O96), MAPK3 (PDB ID: 4QTB), CDK4 (PDB ID: 9CSK), CDK6 (PDB ID: 5L2T), JAK1 (PDB ID: 6N7A), JAK2 (PDB ID: 3E64), CTNNB1 (PDB ID: 7ZRB), PIK3CA (PDB ID: 4JPS), SRC (PDB ID: 7NG7), and STAT3 (PDB ID: 6NUQ) were retrieved from the RCSB Protein Data Bank (https://www.rcsb.org/).

UCSF Chimera 1.18 software was used to preprocess receptor proteins by removing ligands and water molecules. Subsequent preparation steps included hydrogenation, charge calculation and energy minimization. The energy minimization process targeted the nearest local minima using the Amber ff4SB force field with 100 steepest descent steps, resulting in the finalized PDB structures for virtual screening.

For the phytochemicals (ligands), the majority of their 3D structures were obtained from the PubChem database (https://pubchem.ncbi.nlm.nih.gov/) and the ChEMBL database (https://www.ebi.ac.uk/chembl/) in SDF format. For phytochemicals not available in these databases, 3D structures were generated using OpenBabel software from 2D structures drawn with Marvin JS software. The structures were subjected to geometry optimization using the mmff94 force field in OpenBabel (PyRx).

Docking studies were conducted with AutoDock Vina in PyRx, utilizing a Lamarckian genetic algorithm-based scoring function. Docking parameters were set to include the active sites of the target proteins where the compounds bind. Additionally, binding interaction within the active site pocket and bond analysis were thoroughly examined. The grid box was positioned to cover the defined binding site region for each structure (Table 1).

**Table 1:** Docking grid box dimensions used for molecular docking of selected target proteins.

Target Protein	PDB ID	Center Box	Box Dimensions (Angstrom)
AKT1	3O96	X: 7.4403	X: 25.8809
		Y: −11.7282	Y: 38.9588
		Z: 15.7335	Z: 25.7383
MAPK3	4QTB	X: 31.1672	X: 35.0002
		Y: 59.6212	Y: 25.7351
		Z: 53.0255	Z: 36.8772
CDK4	9CSK	X: 35.7463	X: 25.0000
		Y: −12.7805	Y: 32.5969
		Z: 56.4573	Z: 23.1276
CDK6	5L2T	X: 24.8763	X: 17.0867
		Y: 35.5077	Y: 19.6339
		Z: −12.8315	Z: 19.4951
JAK1	6N7A	X: 11.9741	X: 23.7758
		Y: 22.2406	Y: 27.6111
		Z: 21.5779	Z: 19.1038
JAK2	3E64	X: 30.4745	X: 27.1267
		Y: 37.5160	Y: 26.0302
		Z: 36.6042	Z: 21.5813
CTNNB1	7ZRB	X: 14.3087	X: 22.8548
		Y: −9.3458	Y: 15.9389
		Z: 5.9199	Z: 20.2439
PIK3CA	4JPS	X: 34.3113	X: 27.9477
		Y:41. 2421	Y: 33.2302
		Z: 38.1552	Z: 29.0969
SRC	7NG7	X: −14.7665	X: 23.9131
		Y: −0.8430	Y: 24.8938
		Z: −10.5355	Z: 17.1088
STAT3	6NUQ	X: 19.1600	X: 33.1712
		Y: 14.1859	Y: 27.6823
		Z: 30.4825	Z: 25.0085

### Docking validation

The accuracy and reproducibility of the docking procedure were validated through redocking. For all ten protein targets, the same PDB structures used in the primary docking analysis were employed for validation by removing their co-crystallized ligands and redocking them into their respective binding sites.

All proteins were prepared by removing water molecules, adding hydrogens, assigning Gasteiger charges, optimizing side-chain geometry, and defining binding grids centered on the coordinates of the crystallized ligand. Co-crystallized ligands were extracted, energy-minimized, and prepared using the same torsional and optimization parameters applied to the compounds from the main docking workflow. Redocking was conducted using the same protocol followed in the primary docking analysis, including the same grid parameters. Root mean square deviation (RMSD) values between the coordinates of the experimental crystallized ligands and the redocked ligands were computed by heavy-atom structural alignment using a nearest-neighbor protocol. An RMSD value below 2 Å was considered a successful reproduction of the experimental pose, ensuring the protein structure was not significantly altered during preparation.

### Molecular dynamics (MD) simulation

Molecular dynamics simulations studies were conducted to further substantiate the docking results. All docking scores for the target proteins adhered to a cutoff of −7.0 kcal/mol. In addition to this cutoff, consideration was also given to thymoquinone, a previously reported major anticancer compound.

Molecular dynamics simulations were conducted using GROMACS 2022.4 software to analyze the interaction and stability of target proteins with phytochemicals. The docked complexes with the best pose based on the docking scores were subjected to MD simulations. The CHARMM27 force field was applied to model the receptor proteins and compounds, and the ligand topologies were generated using the SwissParam web server (https://old.swissparam.ch/).

A triclinic box was configured for all systems with a minimum distance of 1.0 nm between the protein complex and the box edge. Systems were solvated using the TIP3P water model and neutralized with appropriate sodium and chloride ions. Energy minimization was performed using the steepest descent algorithm. Equilibration phases were carried out under the Canonical ensemble (NVT) and isothermal-isobaric ensemble (NPT) conditions at 310 K and 1 bar for 100 ps.

A production run of 100 ns was performed for each system. All trajectories obtained from the simulations were analyzed by RMSD and root mean square fluctuation (RMSF).

### Preparation of the sample for GC-MS and *in vitro* antiproliferative assay

The polyherbal formulation, comprised of aforementioned plant materials, was prepared using the supercritical carbon dioxide (CO_2_) method at 42 °C, under 30 MPa pressure at a flow rate of 4 mL/min for 1 h [16]. Vernolac extract from Batch No. V24085 was used for both GC-MS analysis and the SRB assay.

### GC-MS analysis

An Agilent GC-MS system (7890A GC, 5975C MS; and 7890B auto sampler) (Agilent Technologies, Inc., Santa Clara, CA, USA) equipped with a J&W DB-5 MS capillary column (5% phenyl methyl siloxane 30.0 m × 250 μm, film thickness 0.25 μm), was used for chromatographic analysis. Helium (99.999%) was used as the carrier gas at a constant flow rate of 1 mL/min. A 1 μL injection volume in split mode with a split ratio of 1:10 was employed. The ionization voltage was 70 eV, and the injector and detector temperatures were maintained at 260 °C and 320 °C, respectively. The oven temperature program was set as follows: initial temperature, 110 °C (isothermal for 5 min); increased to 280 °C at 20 °C/min (isothermal for 1 min); then further increased to 320 °C at 20 °C/min (isothermal for 5 min). Data acquisition and system control were carried out using MS Solution software. Mass spectral interpretation of the GC–MS data was performed using the National Institute of Standards and Technology (NIST) library.

### Cell culture

All cell lines were cultured and maintained according to the American Type Culture Collection (ATCC) guidelines. The cancer cell lines MCF-7 (ATCC HTB-22), Caco-2 (ATCC HTB-37), and NTERA 2 cl.D1 (ATCC CRL-1973) were cultured in Dulbecco’s Modified Eagle Medium (DMEM), and MCF-10A (ATCC CRL-10317) cells in Mammary Epithelial Cell Basal Medium (MEBM). Fetal bovine serum (FBS) 10%, 50 IU/mL penicillin, and 50 µg/mL streptomycin antibiotic mixtures were added to the culture medium according to ATCC guidelines. All cell lines were maintained in a 95% air and 5% CO_2_ atmosphere with 95% humidity at 37 °C. The cells were maintained by sub-culturing in 25 cm^2^ (T25) cell culture flasks, and cells growing in the exponential phase were used for the SRB assay.

### Sulforhodamine B (SRB) assay

The antiproliferative effect of the Vernolac extract was evaluated using the Sulforhodamine B (SRB) assay, following the protocol described by Rajagopalan et al. [32]. The collected Vernolac extract was dissolved in 0.08% DMSO and serially diluted with media at concentrations ranging from 6.25 to 400 µg/mL. Cells were seeded into 96-well plates (10^4^ cells/well in 100 µL of media) and allowed to attach for 24 h at 37 °C in a humidified atmosphere containing 5% CO_2_. Cells were treated with different concentrations of Vernolac and incubated for 24 h and 48 h. Cells in the control group received only media containing 0.08% DMSO. Following treatment, cells were washed twice with phosphate-buffered saline (PBS), fixed by adding 50% ice-cold trichloroacetic acid (TCA), and incubated at 4 °C for 1 h. The plates were washed with tap water five times and air-dried. Subsequently, 50 µL of 0.4% (w/v) SRB dye prepared in 1% acetic acid was added to each well and incubated in the dark at room temperature for 15 min. Excess dye was removed by washing the wells with 1% acetic acid. The plates were air-dried, and the protein-bound SRB dye was solubilized by adding 100 µL of 10 mM unbuffered Tris base to each well. The plates were then placed on an orbital shaker for 1 h at room temperature. The absorbance was recorded at 540 nm using a Synergy HT microplate reader, BioTek Instruments, USA, and the percentage cell viability was calculated using the following formula: Percentage cell viability = [(A_s_ – A_b_)/ (A_c_ – A_b_)] × 100, where A_s_ = absorbance of the sample, A_c_ = absorbance of the negative control, and A_b_ = absorbance of the blank. The IC_50_ values were calculated by non-linear curve fit analysis using GraphPad Prism 8.0.1 (GraphPad Software Inc., San Diego, CA, USA) with R^2^ > 0.9 and p > 0.5. All data were presented as mean ± SD, obtained from three independent replicates (n = 3) for each cell line.

## Results

### Screening of phytochemicals of Vernolac

**Fig 2:**
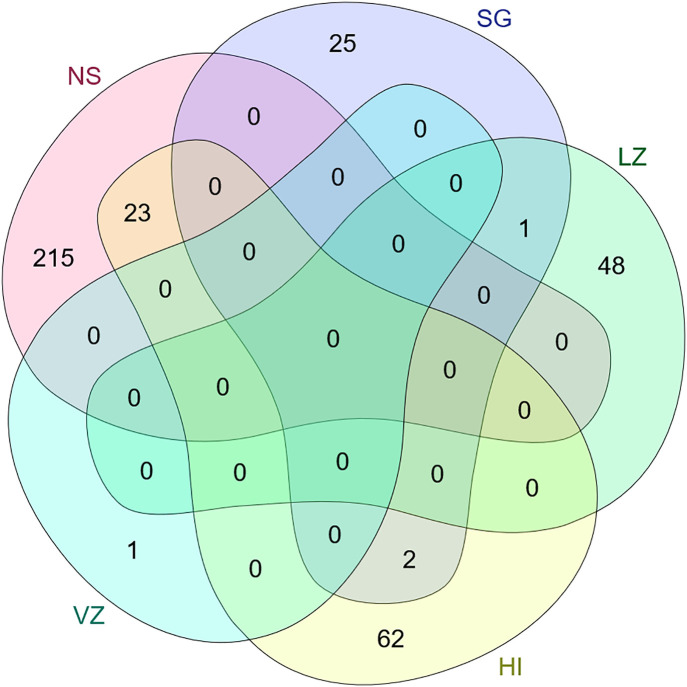
Venn diagram representing the distribution of phytochemicals among the five herbs in Vernolac. NS- *Nigella sativa*, SG- *Smilax glabra*, LZ- *Leucas zeylanica*, HI- *Hemidesmus indicus*, VZ- *Vernonia zeylanica.*

After an extensive search of compounds of Vernolac through the IMPAAT database and literature mining, 403 compounds were retrieved, including 238, 28, 49, 01, and 87 compounds in *N. sativa, S. glabra, L. zeylanica, V. zeylanica, and H. indicus*, respectively. Some herbs shared similar compounds (Fig 2). The overlapping regions indicate the presence of shared phytochemicals in all five herbs, suggesting possible synergistic effects that may contribute to the formulation’s overall anticancer potential.

### Drug likeness and oral bioavailability of Vernolac compounds

A total of 489 compounds in Vernolac obtained through literature and database mining were screened for drug-likeness and oral bioavailability using SwissADME. Based on Lipinski’s rule of five (≤2 violations) and a bioavailability score >0.30, 155 compounds were identified as drug-like and prioritized for further analyses.

### Target prediction of compounds and cancer

Target prediction for drug-like compounds selected from SwissADME predictions was performed, obtaining 500, 427, 629, 422, and 29 targets for *N. sativa*, *S. glabra*, *L. zeylanica*, *H. indicus*, and *V. zeylanica*, respectively. After removing duplicates, a total of 927 targets were obtained. To identify cancer targets, a search was conducted in the GeneCards database, obtaining 18,803 cancer-related protein-coding genes.

### Acquisition of common targets

**Fig 3:**
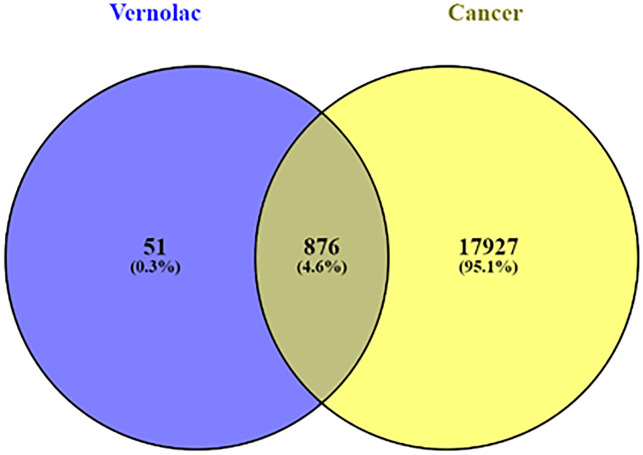
Venn diagram illustrating the intersection between predicted targets of Vernolac and cancer targets.

Using Venny 2.1.0, potential Vernolac targets relevant to cancer were identified by intersecting them with cancer-related targets. A total of 876 targets associated with both Vernolac and cancer were identified (Fig 3).

### Core target selection for further analyses

**Fig 4:**
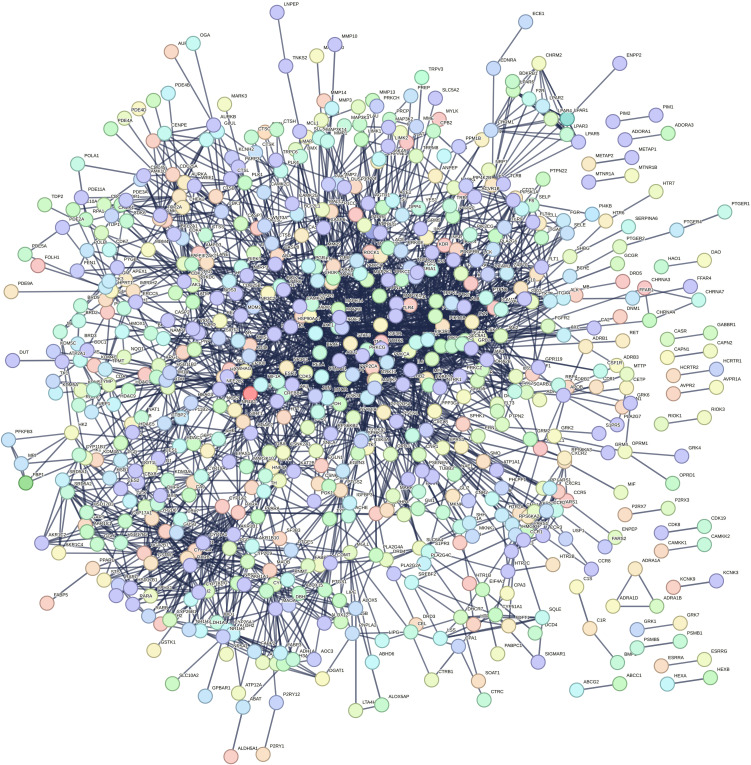
Preliminary protein-protein interaction (PPI) network. Nodes- proteins, Edges- interactions.

A stepwise filtering strategy was applied to extract the core targets from the PPI network, prioritizing highly connected and biologically significant nodes. To ensure the inclusion of only strongly supported protein-protein interactions, the initial PPI network was constructed using high-confidence interactions (confidence score >0.9) from the STRING database. This resulted in a network consisting of 679 nodes and 4934 edges. Disconnected nodes were hidden in the preliminary PPI network depicted in Fig 4.

To further refine the network and select core proteins, the DC topology parameter was applied as the primary filtering criterion. DC is the number of edges/interactions of a particular node. Nodes with high DC values represent hub proteins that interact significantly with many other proteins in the network, indicating their potential importance in cellular processes. Therefore, targets with DC ≥ 20 were selected. After applying this threshold, the number of targets was reduced to 137 nodes, representing central nodes located at the center of the network. These 137 core targets were selected for further analyses, including topology analysis, clustering, pathway enrichment, and hub node identification, to explore their role in the molecular mechanisms.

### Core target PPI network

**Fig 5:**
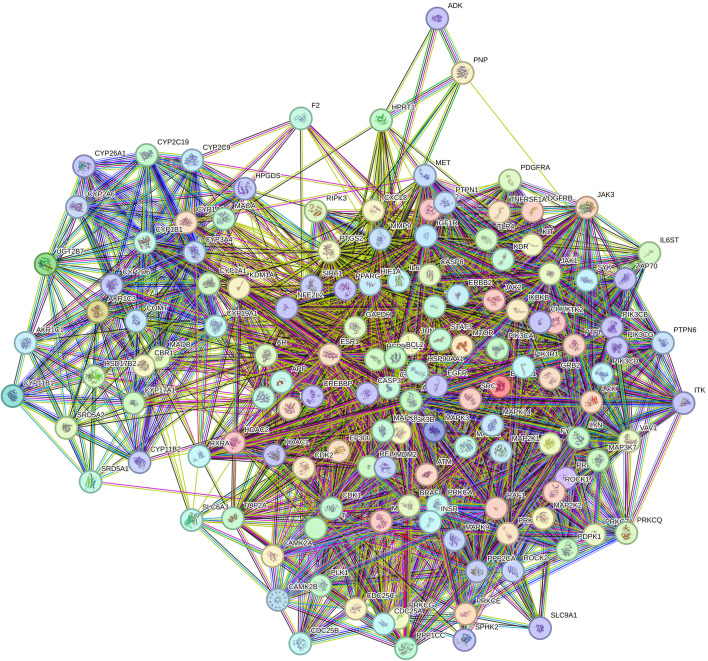
Core target protein-protein interaction network. Nodes represent target proteins. Interactions between proteins are presented by edges.

The core target PPI network was constructed using the STRING database with 137 key targets (Fig 5). The resulting network consisted of 137 nodes and 3183 edges. According to the network statistics, the average node degree and average local clustering coefficient were 46.5 and 0.648, respectively. The PPI enrichment p-value was < 1.0e-16, indicating that the observed interactions were significantly more than expected by random chance and a strong functional association among the targets.

Moreover, the phytochemicals targeting core 137 targets are provided in S1 Table, including their PubChem ID, molecular weight, and druglikeness properties (Lipinski rule).

### Topological analysis of the PPI network

The key nodes with DC > 100 are presented in Table 2, including their respective BC and CC values. According to the CytoNCA analysis, the top 20 nodes include AKT1, SRC, EGFR, STAT3, CTNNB1, HSP90AA1, ESR1, JUN, MAPK1, BCL2, CASP3, IL6, MAPK3, GAPDH, TNF, PIK3CA, HSP90AB1, PIK3R1, and JAK2. Results of CytoNCA topology analysis of 137 core target proteins are provided in the S2 Table.

**Table 2:** CytoNCA topology analysis of the top 20 core target proteins.

Target	Degree centrality	Betweenness centrality	Closeness centrality
AKT1	154.96	1766	0.6031069
SRC	151.38	449	0.59821291
EGFR	151.24	134	0.60475951
STAT3	148.51	28	0.59421991
CTNNB1	140.32	934	0.57684926
HSP90AA1	136.88	226	0.56991102
ESR1	133.19	1334	0.58049449
JUN	131.76	264	0.56165885
MAPK1	129.59	0.5	0.55806456
BCL2	128.02	248	0.5489157
CASP3	125.30	65.5	0.54274189
IL6	123.82	325	0.53371403
MAPK3	123.47	33	0.5512886
GAPDH	123.12	913	0.54348646
TNF	122.80	528.5	0.52283423
PIK3CA	120.52	14	0.52999167
HSP90AB1	117.66	68	0.53992448
PIK3R1	109.57	221	0.52096769
JAK2	109.47	70	0.49898347
MTOR	108.27	36	0.50260174

In addition to degree centrality, closeness centrality (CC) and betweenness centrality (BC) were analyzed to further evaluate the significance of nodes in the network. Closeness centrality (CC) is defined as the reciprocal of the average length of the shortest paths between a node and all other nodes in the network. Higher CC values indicate that a node is more centrally located, allowing for faster information or signal transmission across the network. Betweenness centrality (BC) measures how often a node appears on the shortest paths between other node pairs in the network. A higher BC value suggests that the node acts as a critical bridge in the network, playing a key role in signal transduction and molecular interactions.

### Identification of hub nodes

**Fig 6:**
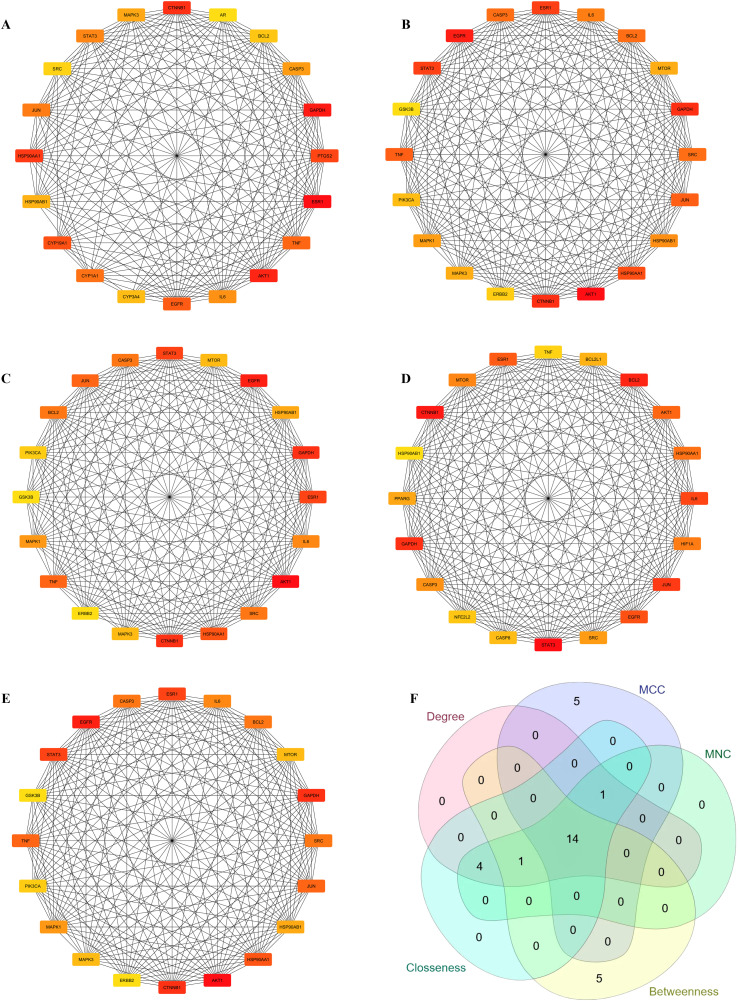
Key subnetworks of the top 20 hub nodes identified using five different algorithms in CytoHubba and their intersection. **(A)** Betweenness; **(B)** Closeness; **(C)** Degree; **(D)** MCC; **(E)** MNC; **(F)** The intersection of five methods. The color intensity of nodes represents the degree of interaction (red = highest score, yellow = lowest score). Rectangles and edges represent proteins and protein-protein interactions, respectively.

To identify hub nodes in the PPI network, a multi-method approach was applied using the CytoHubba plug-in. Five different methods in the CytoHubba, including MCC, closeness, MNC, degree, and betweenness, were used to rank nodes. As Fig 6A–6E shows, the top 20 nodes were selected for each method. Table 3 summarizes the top 20 nodes with scores for each method. The top-ranked nodes from all five methods were compared using a Venn diagram (Fig 6F) to identify hub nodes. The intersection of five methods resulted in the identification of 14 hub proteins that were common across all methods. The 14 intersecting hub proteins include, AKT1, BCL2, CASP3, CTNNB1, EGFR, ESR1, GAPDH, HSP90AA1, HSP90AB1, IL6, JUN, SRC, STAT3, and TNF. These proteins are highly connected within the network, indicating their functional significance and potential role as key regulators in cancer-related pathways.

**Table 3:** Top 20 hub proteins identified using five centrality algorithms in CytoHubba.

Gene name	Degree score	Gene name	MCC score	Gene name	MNC Score	Gene name	Betweenness score	Gene name	Closeness score
AKT1	103	STAT3	1.48E + 30	AKT1	103	ESR1	808.28724	AKT1	119.5
EGFR	97	CTNNB1	1.48E + 30	EGFR	97	GAPDH	716.90958	EGFR	116.5
CTNNB1	95	BCL2	1.48E + 30	CTNNB1	95	AKT1	665.09285	CTNNB1	115.5
GAPDH	95	GAPDH	1.48E + 30	GAPDH	95	CTNNB1	462.56685	GAPDH	115.5
STAT3	94	JUN	1.48E + 30	STAT3	94	HSP90AA1	394.74473	STAT3	115
ESR1	94	IL6	1.48E + 30	ESR1	94	PTGS2	394.60535	ESR1	115
HSP90AA1	93	EGFR	1.48E + 30	HSP90AA1	93	CYP19A1	367.82061	HSP90AA1	114.5
TNF	92	ESR1	1.48E + 30	TNF	92	EGFR	364.22511	TNF	114
JUN	92	AKT1	1.48E + 30	JUN	92	TNF	327.96152	JUN	114
CASP3	91	HSP90AA1	1.48E + 30	CASP3	91	CYP1A1	325.35134	CASP3	113.5
SRC	91	HIF1A	1.48E + 30	SRC	91	JUN	319.30351	SRC	113.5
BCL2	91	MTOR	1.48E + 30	BCL2	91	STAT3	305.42203	BCL2	113.5
IL6	90	CASP3	1.48E + 30	IL6	90	IL6	304.18051	IL6	113
MAPK1	84	SRC	1.48E + 30	MAPK1	84	CASP3	292.08495	HSP90AB1	109.5
HSP90AB1	83	PPARG	1.48E + 30	HSP90AB1	83	MAPK3	266.33965	MAPK1	109.5
MAPK3	82	BCL2L1	1.47E + 30	MAPK3	82	HSP90AB1	251.31164	MAPK3	109
MTOR	82	CASP8	1.43E + 30	MTOR	82	CYP3A4	244.93746	MTOR	109
PIK3CA	77	NFE2L2	1.42E + 30	PIK3CA	77	BCL2	244.73445	PIK3CA	106.5
GSK3B	74	TNF	1.37E + 30	GSK3B	74	SRC	244.37431	ERBB2	105
ERBB2	74	HSP90AB1	1.30E + 30	ERBB2	74	AR	212.75465	GSK3B	104.5

### Identification of core clusters

**Fig 7:**
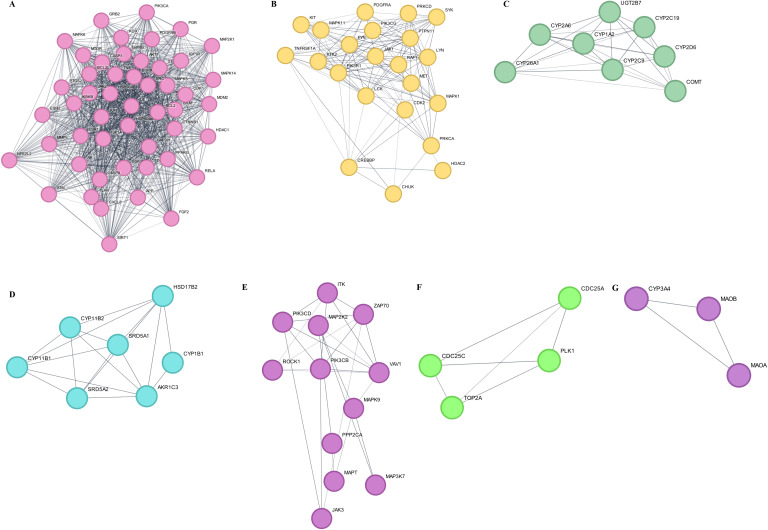
Clustering of the protein–protein interaction (PPI) network using the MCODE analysis. **(A)** Cluster 1, **(B)** Cluster 2, **(C)** Cluster 3, **(D)** Cluster 4, **(E)** Cluster 5, **(F)** Cluster 6, **(G)** Cluster. Colored circles and edges represent the proteins and protein-protein interactions, respectively.

The MCODE analysis identified 7 clusters within the core PPI network, highlighting densely connected functional clusters (Fig 7). Each cluster represents potential functional modules associated with key biological processes. Results of MCODE analysis, including MCODE status, MCODE score, and degree, are presented in the S3 Table.

### Formula-Herb-Compound-Target-Disease network construction and analysis

**Fig 8:**
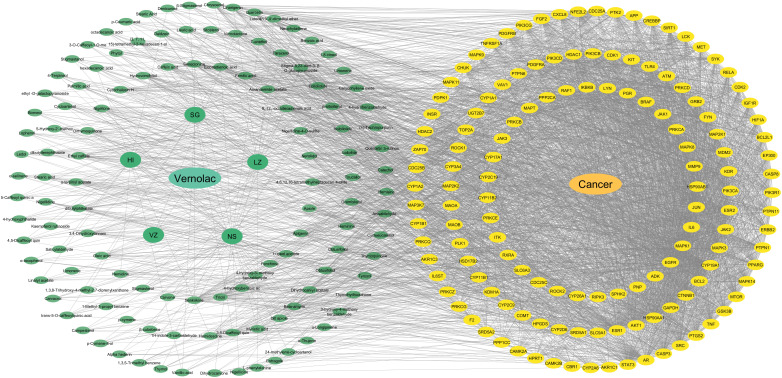
Formula-Herb-Compound-Target-Disease network. The central green ellipse represents the Vernolac formula, connected to green circles (herbs) and surrounding green ellipses (compounds). Yellow ellipses indicate cancer (center) and associated targets (outer rings), illustrating the multi-component, multi-target nature of Vernolac in cancer therapy. Edges represent interactions between each component in the network.

A network was constructed using Cytoscape 3.10.3 to visualize interactions between herbs, compounds, and cancer-related targets. The network comprised 265 nodes and 4709 edges (Fig 8). The topology analysis of the network revealed that one compound can target multiple genes, and one gene can be targeted by multiple compounds. Among all the compounds, the top 10 with the highest degree were carvacrol, cycloartenol, 24-methylene-cycloartanol, 4-terpineol, campesterol, cycloeucalenol, dithymoquinone, gramisterol, lophenol, and nigellicine, highlighting their significant involvement in the regulation of multiple cancer-related targets. The AR was the gene associated with the highest number of compounds, followed by high-degree targets with degree ≥90, including CYP19A1, JAK1, JAK2, PRKCA, SLC6A3, CYP2C19, PTPN1, ESR1, UGT2B7, PTGS2, CYP17A1, and ESR2. As the network illustrates, the interactions between compounds of Vernolac and cancer-related targets signify the therapeutic effect of Vernolac through synergistic actions of multiple compounds and targets.

### GO term enrichment analysis

**Fig 9:**
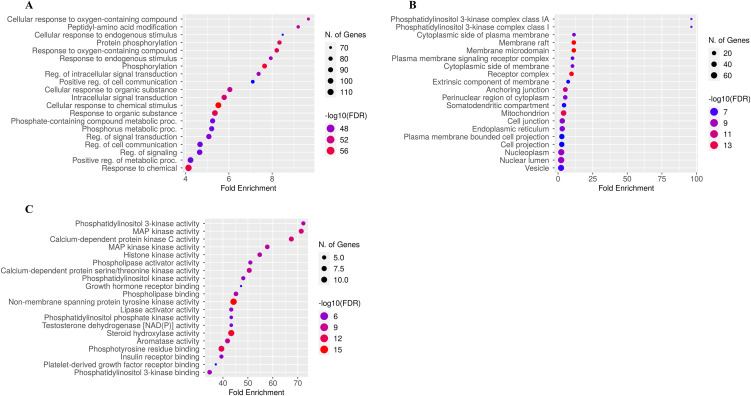
GO enrichment analysis of core targets. **(A)** GO biological process terms. **(B)** GO cellular process terms. **(C)** GO molecular function terms. The size of each dot corresponds to the number of genes annotated in the entry. The color of each dot corresponds to the false discovery rate (FDR).

In the GO term enrichment analysis, a total of 1000 BP terms, 276 CC terms, and 523 MF terms were identified. The top 20 enriched terms in each category are presented in Fig 9 and Table 4. According to the results, the BP terms were mainly related to the immune regulation, cellular responses, metabolic pathways, cellular transport processes, and cellular differentiation. The enriched CC terms highlight membrane-bound signaling complexes, cytoplasmic structures, chromatin regulators, and neuronal components. The MF GO terms are primarily enriched in kinase activity, signal transduction, receptor interactions, and enzymatic activities.

**Table 4:** GO enrichment analysis.

Category	Term	Genes	Count	Enrichment FDR
Biological processes	GO:0060439 – Trachea morphogenesis	MAPK1, MAPK3, MAP2K2, CTNNB1, MAP2K1	5	2.04E-08
	GO:0097267 – Omega-hydroxylase P450 pathway	CYP1B1, CYP2C9, CYP1A1, CYP1A2, CYP2C19	5	2.04E-08
	GO:0002223 – Stimulatory C-type lectin receptor signaling pathway	SYK, CREBBP, FYN, EP300, IKBKB, MAP3K7, SRC, LYN	8	2.45E-12
	GO:1990840 – Response to lectin	SYK, CREBBP, FYN, EP300, IKBKB, MAP3K7, SRC, LYN	8	2.45E-12
	GO:1990858 – Cellular response to lectin	SYK, CREBBP, FYN, EP300, IKBKB, MAP3K7, SRC, LYN	8	2.45E-12
	GO:0060020 – Bergmann glial cell differentiation	MAPK1, MAPK3, MAP2K1, PTPN11	4	1.88E-06
	GO:0097709 – Connective tissue replacement	HIF1A, PPARG, ROCK1, ROCK2	4	1.88E-06
	GO:0048308 – Organelle inheritance	MAPK1, MAPK3, CDK1, MAP2K2, MAP2K1	5	1.16E-07
	GO:0048313 – Golgi inheritance	MAPK1, MAPK3, CDK1, MAP2K2, MAP2K1	5	1.16E-07
	GO:0019373 – Epoxygenase P450 pathway	CYP2C9, CYP2C19, CYP2A6, CYP1B1, CYP1A1, CYP1A2	6	6.93E-09
	GO:0070102 - Interleukin-6-mediated signaling pathway	IL6ST, IL6, STAT3, SRC, JAK2, JAK1	6	6.93E-09
	GO:0035994 – Response to muscle stretch	MAPK14, PIK3CA, RAF1, RELA, JUN, SLC9A1, CTNNB1, PTK2	8	1.66E-11
	GO:1904385 – Cellular response to angiotensin	CAMK2A, PRKCD, NFE2L2, IGF1R, SRC, ROCK1, RELA, ROCK2	8	1.66E-11
	GO:1990776 – Response to angiotensin	CAMK2A, PRKCD, ROCK1, PTGS2, NFE2L2, IGF1R, SRC, RELA, ROCK	9	9.60E-13
	GO:0038095 – Fc-epsilon receptor signaling pathway	MAPK9, PRKCQ, IKBKB, MAPK8, MAP3K7, VAV1, SYK, LYN	8	2.30E-11
	GO:0060396 – Growth hormone receptor signaling pathway	JAK2, JAK3, JAK1, STAT3, PIK3R1, PTK2, PTPN1, LYN	8	2.30E-11
	GO:0071378 – Cellular response to growth hormone stimulus	JAK2, JAK3, JAK1, STAT3, PIK3R1, PTK2, PTPN1, LYN	8	2.30E-11
	GO:2000641 – Reg. of early endosome to late endosome transport	MAPK1, MAPK3, SRC, MAP2K2, MAP2K1	5	2.23E-07
	GO:0002433 – Immune response-regulating cell surface receptor signaling pathway in	FYN, VAV1, PRKCD, SYK, PTK2, PRKCE, SRC, LYN	8	3.13E-11
	GO:0038096 – Fc-gamma receptor signaling pathway involved in phagocytosis	FYN, VAV1, PRKCD, SYK, PTK2, PRKCE, SRC, LYN	8	3.13E-11
Cellular components	GO:0099091 – Postsynaptic specialization intracellular component	CTNNB1, SRC, LYN	3	0.000201
	GO:0008385 – Kappa kinase complex	IKBKB, CHUK, MAP3K7	3	0.00033
	GO:0044292 – Dendrite terminus	HSP90AA1, HSP90AB1, SRC	3	0.0004
	GO:0042101 – T cell receptor complex	ZAP70, SYK, PTPN6	3	0.000707
	GO:0035631 - CD40 receptor complex	IKBKB, CHUK	2	0.007999
	GO:0005942 – Phosphatidylinositol 3-kinase complex	PIK3CB, PIK3CG, PIK3CA, PIK3R1, PIK3CD	5	8.38E-06
	GO:0031143 – Pseudopodium	MAPK1, MAPK3, RAF1	3	0.000972
	GO:0030877 – β -catenin destruction complex	GSK3B, CTNNB1	2	0.009333
	GO:0099522 – Cytosolic region	PRKCG, PRKCB	2	0.013034
	GO:1990909 – Wnt signalosome	CTNNB1, GSK3B	2	0.013034
	GO:0016581 – NuRD complex	HDAC1, HDAC2	2	0.014363
	GO:0090545 – CHD-type complex	HDAC1, HDAC2	2	0.014363
	GO:0097524 – Sperm plasma membrane	HSP90AA1, HSP90AB1	2	0.014363
	GO:0035098 ESC/E(Z) complex	SIRT1, HDAC2	2	0.015598
	GO:0044214 – Spanning component of plasma membrane	EGFR, ERBB2	2	0.015598
	GO:0044305 – Calyx of Held	PRKCG, PRKCB	2	0.015598
	GO:0043209 – Myelin sheath	HSP90AA1, PRKCZ, ERBB2, SRD5A1, BCL2	5	6.28E-05
	GO:0097038 – Perinuclear endoplasmic reticulum	PIK3R1, FYN	2	0.020688
	GO:0000791 – Euchromatin	ESR1, SIRT1, CTNNB1, JUN, JAK2, HIF1A	6	1.81E-05
	GO:0005901 – Caveola	INSR, SRC, PTGS2, JAK2, MAPK1, MAPK3, IGF1R, CTNNB1	8	1.04E-06
Molecular functions	GO:0035004 – Phosphatidylinositol 3-kinase activity	PIK3CB, PIK3CG, PIK3CA, PIK3CD, ATM	5	4.31E-08
	GO:0004707 – MAP kinase activity	MAPK9, MAPK1, MAPK3, MAPK8, MAPK14, MAPK11, MAP3K7	7	4.96E-11
	GO:0004698 – Calcium-dependent protein kinase C activity	PRKCQ, PRKCZ, PRKCG, PRKCA, PRKCD, PRKCB, PRKCE	7	7.73E-11
	GO:0004708 – MAP kinase activity	MAP2K2, MAP2K1, MAPK1, MAPK3, BRAF, MAPK14	6	6.61E-09
	GO:0035173 – Histone kinase activity	JAK2, CDK2, PRKCA, PRKCB, CDK1, ATM	6	9.30E-09
	GO:0016004 – Phospholipase activator activity	FYN, LCK, SRC, CASP3, PDPK1	5	2.99E-07
	GO:0009931 – Calcium-dependent protein serine/threonine kinase activity	PRKCQ, PRKCZ, PRKCG, PRKCA, PRKCD, PRKCB, PRKCE	7	7.84E-10
	GO:0052742 – Phosphatidylinositol kinase activity	PIK3CB, PIK3CG, PIK3CA, PIK3CD, ATM	5	3.87E-07
	GO:0005131 – Growth hormone receptor binding	JAK2, JAK3, JAK1	3	0.000162
	GO:0043274 – Phospholipase binding	PRKCZ, FYN, PDPK1, SYK, LCK, SRC	6	3.04E-08
	GO:0004715 – Non-membrane spanning protein tyrosine kinase activity	FYN, JAK2, JAK3, ITK, ZAP70, JAK1, SYK, PTK2, LCK, SRC, LYN, PRKCD	12	7.70E-16
	GO:0060229 – Lipase activator activity	FYN, LCK, SRC, CASP3, PDPK1	5	6.70E-07
	GO:0016307 – Phosphatidylinositol phosphate kinase activity	PIK3CB, PIK3CG, PIK3CA, PIK3CD	4	1.19E-05
	GO:0030283 – Testosterone dehydrogenase [NAD(P)] activity	HSD17B2, SRD5A2, AKR1C1, AKR1C3	4	1.19E-05
	GO:0008395 – Steroid hydroxylase activity	CYP2C9, CYP17A1, CYP3A4, CYP11B1, CYP2C19, CYP11B2, CYP2A6, CYP1B1, CYP1A1	9	1.97E-14
	GO:0070330 – Aromatase activity	CYP19A1, CYP1B1, CYP2C9, CYP1A1, CYP1A2, CYP3A4, CYP2C19	7	3.14E-09
	GO:0001784 – Phosphotyrosine residue binding	MAPK1, MAPK3, PTPN6, ZAP70, GRB2, SYK, PTPN11, VAV1, PIK3R1, LCK	10	1.01E-12
	GO:0005158 – Insulin receptor binding	IGF1R, PTPN1, SRC, PIK3R1, PTPN11	5	1.09E-06
	GO:0005161 – Platelet-derived growth factor receptor binding	LYN, PDGFRB, PDGFRA	3	0.000334
	GO:0043548 – Phosphatidylinositol 3-kinase binding	JAK2, PDGFRB, IGF1R, PIK3R1, INSR, LCK	6	1.54E-07

### KEGG pathway enrichment analysis

**Fig 10:**
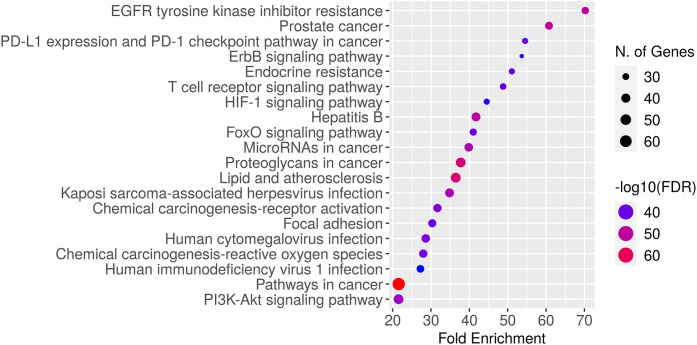
KEGG pathway enrichment analysis. The size of each dot corresponds to the number of genes annotated in the entry. The color of each dot corresponds to the false discovery rate (FDR).

The 137 common targets were input into ShinyGO for KEGG pathway enrichment analysis to explore the critical signaling pathways of Vernolac targeting cancer. A total of 217 KEGG pathways were significantly enriched. The top 20 KEGG pathways were selected based on fold enrichment and FDR, and they were primarily related to cancer, immune regulation, endocrine pathways, and metabolic pathways (Table 5). The dot plot of the top 20 KEGG pathways is shown in Fig 10. Notably, these pathways signify Vernolac’s potential role in targeting various types of cancers, such as prostate cancer, glioma, non-small cell lung carcinoma, endometrial cancer, bladder cancer, pancreatic cancer, renal cell carcinoma, as well as hematological malignancies, including acute myeloid leukemia and chronic myeloid leukemia.

**Table 5:** Top 20 KEGG pathways.

Pathway	Genes	Count	Enrichment FDR
Path:hsa01521 – EGFR tyrosine kinase inhibitor resistance	EGFR, ERBB2, AKT1, FGF2, MTOR, GRB2, GSK3B, IGF1R, IL6, JAK1, JAK2, KDR, MET,PDGFRA, PDGFRB, PIK3CA, PIK3CB,PIK3CD, PIK3R1, PRKCA, PRKCB, PRKCG, MAPK1, MAPK3, MAP2K1, MAP2K2, RAF1, BCL2, BCL2L1, SRC, BRAF, STAT3	32	1.63E-50
Path:hsa05215 – Prostate cancer	CDK2, CHUK, CREBBP, CTNNB1, EGFR, EP300, ERBB2, AKT1, MTOR, GRB2, GSK3B, HSP90AA1, HSP90AB1, IGF1R, IKBKB, AR, MDM2, MMP9, PDGFRA, PDGFRB, PDPK1, PIK3CA, PIK3CB, PIK3CD, PIK3R1, MAPK1, MAPK3, MAP2K1, MAP2K2, RAF1, BCL2, RELA, SRD5A2, BRAF	34	4.34E-51
Path:hsa04370 – VEGF signaling pathway	MAPK14, AKT1, KDR, PIK3CA, PIK3CB, PIK3CD, PIK3R1, PRKCA, PRKCB, PRKCG, MAPK1, MAPK3, MAPK11, MAP2K1, MAP2K2, SPHK2, PTGS2, PTK2, RAF1, SRC	20	4.61E-30
Path:hsa04917 – Prolactin signaling pathway	MAPK14, CYP17A1, AKT1, ESR1, ESR2, GRB2, GSK3B, JAK2, PIK3CA, PIK3CB, PIK3CD, PIK3R1, MAPK1, MAPK3, MAPK8, MAPK11, MAPK9, MAP2K1, MAP2K2, RAF1, RELA, SRC, STAT3	23	5.76E-34
Path:hsa05235 – PD-L1 expression and PD-1 checkpoint pathway in cancer	CHUK, MAPK14, EGFR, AKT1, MTOR, HIF1A, IKBKB, JAK1, JAK2, JUN, LCK, PIK3CA, PIK3CB, PIK3CD, PIK3R1, PRKCQ, MAPK1, MAPK3, MAPK11, MAP2K1, MAP2K2, PTPN6, PTPN11, RAF1, RELA, STAT3, TLR4, ZAP70	28	1.22E-40
Path:hsa04012 – ErbB signaling pathway	EGFR, ERBB2, AKT1, MTOR, GRB2, GSK3B, JUN, PIK3CA, PIK3CB, PIK3CD, PIK3R1, PRKCA, PRKCB, PRKCG, MAPK1, MAPK3, MAPK8, MAPK9, MAP2K1, MAP2K2, PTK2, RAF1, SRC, BRAF, CAMK2A, CAMK2B	26	1.49E-37
Path:hsa04664 – Fc epsilon RI signaling pathway	MAPK14, AKT1, FYN, GRB2, LYN, PDPK1, PIK3CA, PIK3CB, PIK3CD, PIK3R1, PRKCA, MAPK1, MAPK3, MAPK8, MAPK11, MAPK9, MAP2K1, MAP2K2, RAF1, SYK, VAV1	21	1.57E-30
Path:hsa05214 – Glioma	EGFR, AKT1, MTOR, GRB2, IGF1R, MDM2, PDGFRA, PDGFRB, PIK3CA, PIK3CB, PIK3CD, PIK3R1, PRKCA, PRKCB, PRKCG, MAPK1, MAPK3, MAP2K1, MAP2K2, RAF1, BRAF, CAMK2A, CAMK2B	23	3.34E-33
Path:hsa05223 – Non-small cell lung cancer	EGFR, ERBB2, AKT1, GRB2, JAK3, MET, PDPK1, PIK3CA, PIK3CB, PIK3CD, PIK3R1, PRKCA, PRKCB, PRKCG, MAPK1, MAPK3, MAP2K1, MAP2K2, RAF1, RXRA, BRAF, STAT3	22	8.67E-32
Path:hsa04930 – Type II diabetes mellitus	MTOR, IKBKB, INSR, PIK3CA, PIK3CB, PIK3CD, PIK3R1, PRKCD, PRKCE, PRKCZ, MAPK1, MAPK3, MAPK8, MAPK9	14	1.15E-20
Path:hsa04960 – Aldosterone-regulated sodium reabsorption	INSR, PDPK1, PIK3CA, PIK3CB, PIK3CD, PIK3R1, PRKCA, PRKCB, PRKCG, MAPK1, MAPK3	11	2.38E-16
Path:hsa01522 – Endocrine resistance	MAPK14, EGFR, ERBB2, AKT1, ESR1, ESR2, MTOR, GRB2, IGF1R, JUN, MDM2, MMP9, PIK3CA, PIK3CB, PIK3CD, PIK3R1, MAPK1, MAPK3, MAPK8, MAPK11, MAPK9, MAP2K1, MAP2K2, PTK2, RAF1, BCL2, SRC, BRAF	28	9.12E-40
Path:hsa05213 Endometrial cancer	CTNNB1, EGFR, ERBB2, AKT1, GRB2, GSK3B, PDPK1, PIK3CA, PIK3CB, PIK3CD, PIK3R1, MAPK1, MAPK3, MAP2K1, MAP2K2, RAF1, BRAF	17	1.41E-24
Path:hsa05219 Bladder cancer	EGFR, ERBB2, CXCL8, MDM2, MMP9, MAPK1, MAPK3, MAP2K1, MAP2K2, RAF1, SRC, BRAF	12	1.20E-17
Path:hsa04660 T cell receptor signaling pathway	CHUK, MAPK14, AKT1, FYN, GRB2, GSK3B, IKBKB, ITK, JUN, LCK, PDPK1, PIK3CA, PIK3CB, PIK3CD, PIK3R1, PRKCQ, MAPK1, MAPK3, MAPK8, MAPK11, MAPK9, MAP2K1, MAP2K2, PTPN6, RAF1, RELA, MAP3K7, VAV1, ZAP70	29	1.56E-40
Path:hsa05212 Pancreatic cancer	CHUK, EGFR, ERBB2, AKT1, MTOR, IKBKB, JAK1, PIK3CA, PIK3CB, PIK3CD, PIK3R1, MAPK1, MAPK3, MAPK8, MAPK9, MAP2K1, RAF1, RELA, BCL2L1, BRAF, STAT3	21	2.09E-29
Path:hsa05221 Acute myeloid leukemia	CHUK, AKT1, MTOR, GRB2, IKBKB, KIT, PIK3CA, PIK3CB, PIK3CD, PIK3R1, MAPK1, MAPK3, MAP2K1, MAP2K2, RAF1, RELA, BRAF, STAT3	18	3.65E-25
Path:hsa05211 Renal cell carcinoma	CREBBP, EP300, AKT1, GRB2, HIF1A, JUN, MET, PIK3CA, PIK3CB, PIK3CD, PIK3R1, MAPK1, MAPK3, MAP2K1, MAP2K2, PTPN11, RAF1, BRAF	18	4.87E-25
Path:hsa05220 -Chronic myeloid leukemia	CHUK, AKT1, GRB2, HDAC1, HDAC2, IKBKB, MDM2, PIK3CA, PIK3CB, PIK3CD, PIK3R1, MAPK1, MAPK3, MAP2K1, MAP2K2, PTPN11, RAF1, RELA, BCL2L1, BRAF	20	1.30E-27
Path:hsa04662 – B cell receptor signaling pathway	CHUK, AKT1, GRB2, GSK3B, IKBKB, JUN, LYN, PIK3CA, PIK3CB, PIK3CD, PIK3R1, PRKCB, MAPK1, MAPK3, MAP2K1, MAP2K2, PTPN6, RAF1, RELA, SYK, VAV1	21	8.81E-29

### Target-pathway network construction

**Fig 11:**
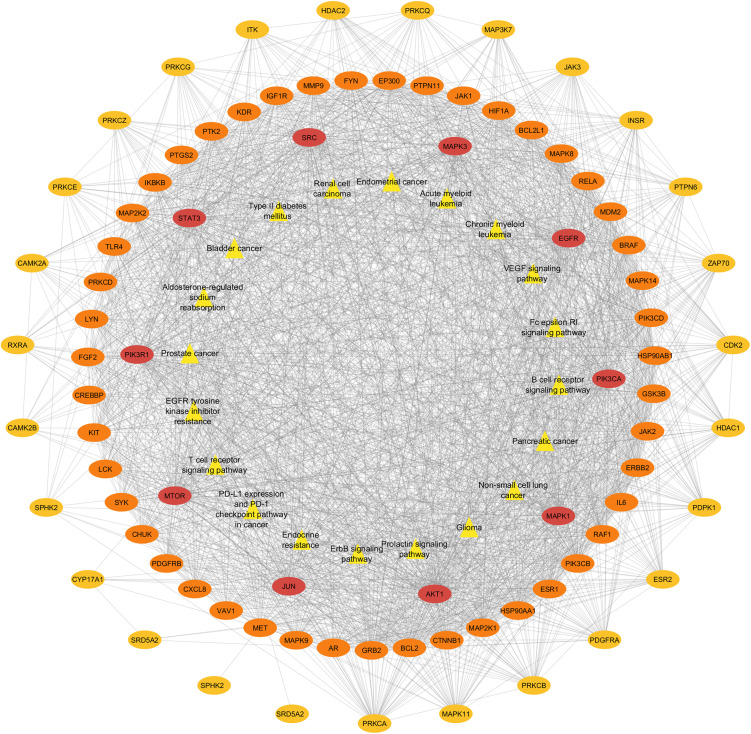
Target-pathway network. Yellow rectangles represent significantly enriched pathways, while elliptical nodes represent target genes. The ellipses are colored from red (innermost, highest degree) to orange and yellow (outermost, lower degree), indicating the decreasing node degree (connectivity) of the targets. Edges represent interactions between target-target and target-pathways. The arrangement highlights central targets with greater involvement across multiple pathways, emphasizing their potential as key regulators in Vernolac’s anticancer activity.

A network was constructed to visualize interactions between the top 20 KEGG pathways and targets (Fig 11). The target-pathway network consisted of 242 nodes and 6804 edges, illustrating the complex interconnectivity between common targets and KEGG pathways. According to the results of network analysis, the top 10 high-degree targets were identified, including AKT1, EGFR, STAT3, CTNNB1, GAPDH, ESR1, TNF, JUN, SRC, and BCL2. The highest degree pathway, representing the most interconnected KEGG pathway, was prostate cancer (hsa05215).

### Molecular docking studies of key target proteins

The ten potential target proteins with high node scores and confidence, including AKT1, CTNNB1, PIK3CA, STAT3, MAPK3, CDK4, CDK6, JAK1, JAK2, and SRC, were selected through network and pathway analysis. Molecular docking studies were conducted using Autodock Vina in PyRx to identify the interactions between the compounds and cancer-related potential target proteins at the molecular level. Table 6 presents the compounds that achieved the highest docking scores with their respective target proteins. The lower the docking score, the higher the binding affinity between the compound and the target protein.

**Table 6:** Docking scores of targets with the corresponding compounds.

Target protein	Compounds		Binding Affinity (Kcal/mol)
1. **RAC-α serine/threonine-protein kinase** (AKT1) [PDB code: 3O96]	Reference	Capiversatib	−10.71
		Dasatinib	−10.35
	Nigellidine		−11.1
	Vernolactone		−9.9
	α- tocopherol		−9.9
	Quercetin		−9.7
	Chryseriol		−9.6
	Tricin		−9.5
	Apigenin		−9.4
	Luteolin		−9.3
	4,8,12,16-tetramethylheptadecan-4-olide		−7.8
	Thymoquinone		−7.0
2. **β-catenin** (CTNNB1) [PDB code: 7ZRB]	Reference	PRI724	−8.1
	α -Hederin		−7.6
	Vernolactone		−7.3
	Thymoquinone		−4.1
3. **Phosphatidylinositol 4,5-bisphosphate 3-kinase catalytic subunit α isoform** (PIK3CA) [PDB code: 4JPS]	Aurantiamide acetate		−7.8
	Vernolactone		−7.1
	Thymoquinone		−6.1
4. **Signal transducer and activator of transcription 3** (STAT3) [PDB code: 6NUQ]	Vernolactone		−7.0
	Thymoquinone		−5.3
5. **Mitogen-activated protein kinase 3** (MAPK3) [PDB code: 4QTB]	α -Hederin		−9.5
	Vernolactone		−9.0
	Thymoquinone		−6.5
6. **Cell division protein kinase 4** (CDK4) [PDB code: 9CSK]	Reference	Ribociclib	−6.5
	α -Hederin		−8.8
	Cytochalasin H		−8.3
	Aurantiamide acetate		−7.3
	Senkirkine		−7.0
	Thymoquinone		−5.4
7. **Cyclin-dependent kinase 6** (CDK6) [PDB code: 5L2T]	Reference	Palbociclib	−10.8
	Apigenin		−9.1
	Luteolin		−8.4
	Chryseriol		−8.3
	Linarigenin		−8.3
	Quercetin		−8.8
	Tricin		−7.9
	α -Hederin		−7.8
	Thymoquinone		−6.5
8. **Tyrosine-protein kinase** JAK1 [PDB code: 6N7A]	Hemidescine		−10.7
	Cytochalasin H		−10.4
	Hemidine		−9.9
	Aurantiamide acetate		−9.1
	β-Sitosterol		−9.1
	Thymoquinone		−6.5
9. **Tyrosine-protein kinase JAK2** [PDB code: 3E64]	Reference	Fedratinib	−10.6
	Hemidine		−9.0
	Hydro vomifoliol		−7.1
	Carvacrol		−7.0
	Dibutyl terephthalate		−7.0
	Thymoquinone		−6.5
	β-Sitosterol		−9.1
10. **Proto-oncogene tyrosine-protein kinase Src** [PDB code: 7NG7]	Reference	Dasatinib	−11.2
	Chryseriol		−8.7
	Linarigenin		−8.7
	Quercetin		−8.6
	Tricin		−7.7
	Cytochalasin H		−8.5
	Thymoquinone		−6.3

Based on the docking scores presented in the Table 6, several key factors stand out regarding the binding affinity of the compounds. While reference compounds such as Palbociclib for CDK6, Fedratinib for JAK2, and Dasatinib for SRC displayed a superior binding affinity compared to the tested compounds, the results also highlight the strong potential of several compounds. For instance, Nigellidine and a group of compounds including α-Hederin, Cytochalasin H, Aurantiamide acetate, and Senkirkine demonstrated superior binding affinity to the reference compounds for AKT1 and CDK4, respectively.

Importantly, a large number of the tested compounds exhibited excellent docking scores, with most compounds achieving a binding affinity of −7 kcal/mol or better. This indicates a strong potential for binding to the target proteins. The compound with the highest overall binding affinity was Nigellidine at −11.1 kcal/mol for AKT1. Other top performers among the compounds included Hemidescine (−10.7 kcal/mol) for JAK1, α -Hederin (−9.5 kcal/mol) for MAPK3, and Apigenin (−9.1 kcal/mol) for CDK6. These results collectively suggest that while some reference controls have the strongest binding, the tested compounds still show promising docking scores and significant potential as inhibitors for the target proteins.

S4 Table presents the bond analysis of the docked compounds in relation to their respective target proteins. For further investigations utilizing molecular dynamic simulations, evaluation criteria encompassed not only docking scores but also the binding interaction within the active site pocket and bond analysis to ensure a comprehensive assessment of molecular stability and interaction strength.

**Fig 12:**
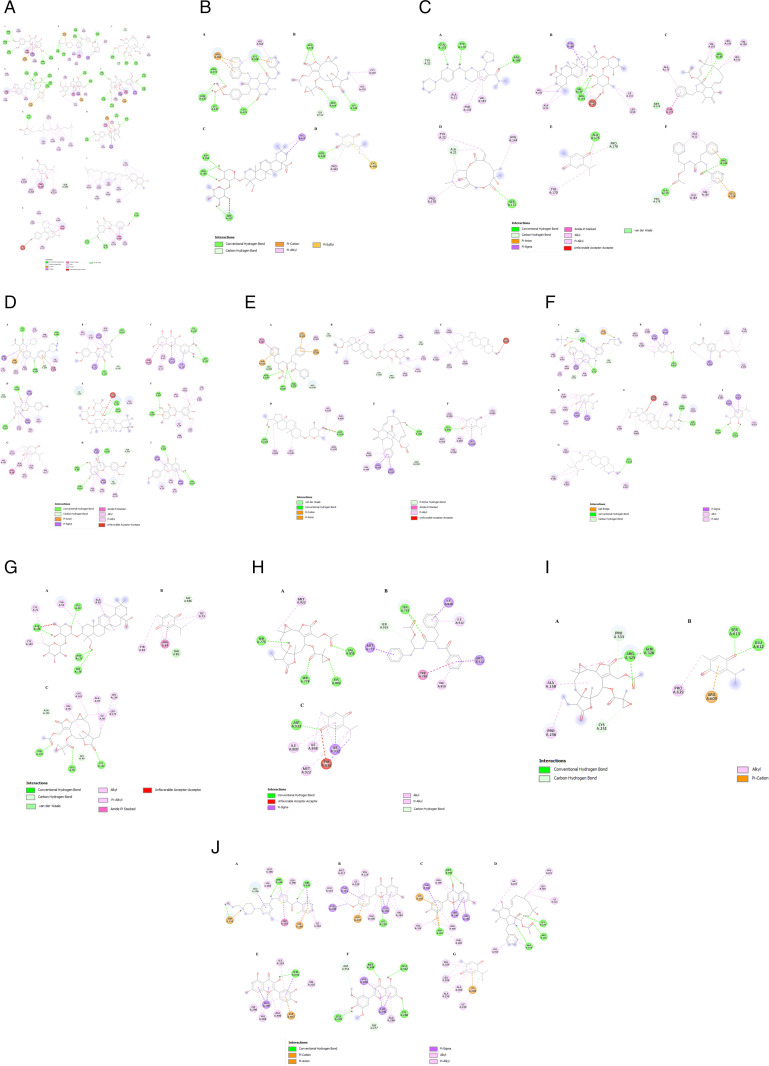
2D protein-ligand interaction diagram. **(1)** 2D protein-ligand interaction of AKT1 protein with compounds. A: Quercetin, B: Dasatinib (reference), C: Vernolactone, D: Capivasertib (reference), E: Tricin, F: Chryseriol, G: 4,8,12,16-Tetramethylheptadecan-4-olide, H: Luteolin, I: Thymoquinone, J: α-tocopherol, K: Nigellidine and L: Apigenin. **(2)** 2D protein-ligand interaction diagram of CTNNB1 protein with compounds; A: PRI724 (reference), B: Vernolactone, C: α-Hederin and D: Thymoquinone. **(3)** 2D protein-ligand interaction diagram of CDK4 protein with compounds; A: Ribociclib (reference), B: α-Hederin, C: Cytochalasin H, D: Senkirkine, E: Thymoquinone and F: Aurantiamide Acetate. **(4)** 2D protein-ligand interaction diagram of CDK6 protein with compounds; A: Palcociclib (reference), B: Chryseriol, C: Quercetin, D: Apigenin, E: α-Hederin, F: Tricin, G: Thymoquinone, H: Luteolin and I: Linarigenin. **(5)** 2D protein-ligand interaction diagram of JAK1 protein with compounds; A: Aurantiamide acetate, B: Hemidescine, C: β-sitosterol, D: Hemidine, E: Cytochalasin H and F: Thymoquinone. **(6)** 2D protein-ligand interaction diagram of JAK2 protein with compounds; A: Fedratinib (reference), B: Carvacrol, C: Dibutyl terephthalate, D: Thymoquinone, E: Hemidine, F: Hydro vomifoliol and G: β-sitosterol. **(7)** 2D protein-ligand interaction diagram of MAPK3 protein with compounds: A: α-Hederin, B: Thymoquinone and C: Vernolactone. **(8)** 2D protein-ligand interaction diagram of PIK3CA protein with compounds; A: Vernolactone, B: Aurantiamide acetate and C: Thymoquinone. **(9)** 2D protein-ligand interaction diagram of STAT3 protein with compounds; A: Vernolactone and B: Thymoquinone. **(10)** 2D protein-ligand interaction diagram of SRC protein with compounds; A: Dasatinib (reference), B: Linarigenin, C: Chryseriol, D: Cytochalasin H, E: Quercetin, F: Tricin and G: Thymoquinone.

The two-dimensional protein–ligand interaction diagrams for each docked target are presented in Fig 12-1–10. The reference compound Capiversatib (Fig 12-1D) primarily relies on hydrogen bonds to interact with AKT1, engaging residues such as THR: A:82, VAL: A:271, TYR: A:272, and ARG: A:273. This indicates a highly specific, polar-driven binding. In contrast, natural compounds such as Nigellidine (Fig 12-1K), Thymoquinone (Fig 12-1I), and Apigenin (Fig 12-1L) demonstrate a greater dependence on hydrophobic interactions with a similar group of residues, including TRP: A:80, LEU: A:264, and VAL: A:270. This suggests that while they may occupy a similar binding pocket, their mode of interaction is fundamentally different.

PRI724, the reference compound for CTNNB1, utilizes a diverse set of interactions, including hydrogen bonds, a hydrophobic interaction with VAL: A:564, and a unique electrostatic interaction with ARG: A:469 (Fig 12-2A). This broad approach allows it to anchor firmly to the protein. The natural compounds, such as α-Hederin and Vernolactone, exhibit a more focused binding profile. α-Hederin (Fig 12-2C) and Thymoquinone (Fig 12-2D) mainly form hydrogen bonds, while Vernolactone (Fig 12-2B) uses a combination of hydrogen bonds and hydrophobic interactions. The reference compound for CDK4, Ribociclib, primarily utilizes hydrophobic interactions with a broad array of residues such as ILE: A:17, VAL: A:25, and LEU: A:152, complemented by a single hydrogen bond with ASP: A:163 (Fig 12-3A). The natural compounds, including α-Hederin (Fig 12-3B), Cytochalasin H (Fig 12-3C), and Asperglaucide (Fig 12-3F), mirror this hydrophobic-dominant strategy. They engage in a similar mix of hydrogen bonds and hydrophobic contacts, indicating that they bind to a similar hydrophobic pocket on the CDK4 protein.

Fedratinib, the reference compound for JAK2, binds through a combination of hydrogen bonds and extensive hydrophobic interactions (Fig 12-6A). The natural compounds like Hemidine (Fig 12-6E), Carvacrol (Fig 12-6B), and Dibutyl terephthalate (Fig 12-6C) primarily rely on hydrophobic interactions with a core set of residues, including LEU: A:855, VAL: A:863, and LEU: A:983. This indicates that they effectively occupy the same hydrophobic pocket as Fedratinib, suggesting a similar mechanism of action.

The reference compound for SRC, Dasatinib, employs a mixed-mode binding strategy, involving hydrogen bonds, hydrophobic interactions, and a few unique electrostatic interactions (Fig 12-10A). The natural compounds, including Chryseriol (Fig 12-10C), Linarigenin (Fig 12-10B), and Quercetin (Fig 12-10E), also employ a blend of hydrogen bonds and hydrophobic interactions. Notably, they often interact with key residues such as MET: A:344 and THR: A:341, which are also crucial for Dasatinib’s binding. This overlap suggests that these natural compounds bind to the same active site and share a common binding pattern with Dasatinib. Additionally, compounds with previously reported anticancer activities were considered for further investigations.

### Docking validation

**Fig 13:**
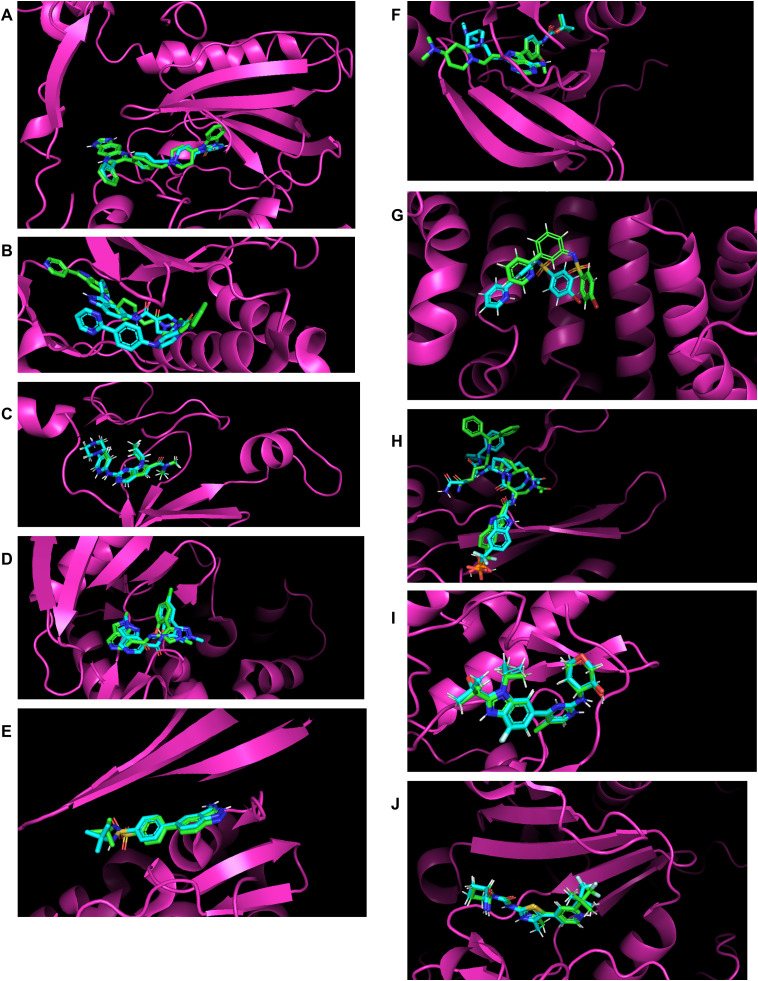
Superimposition of redocked ligands onto the co-crystallized complex in the active site using PyMOL. **A.** AKT1; **B.** MAPK3; **C.** CDK6; **D.** JAK1; **E.** JAK2; **F.** SRC; **G.** CTNNB1; **H.** STAT3; **I.** CDK4; **J.** PIK3CA.

Redocked RMSD values ranged from 0.332 Å to 4.382 Å, reflecting both highly accurate pose recovery and deviations attributable to the intrinsic flexibility of certain targets (Table 7). The structural overlays shown in Fig 13 demonstrate that the redocked ligands align closely with the co-crystallized poses within the active sites of their respective proteins, providing visual confirmation of the accuracy of the docking protocol.

**Table 7:** Structural details and RMSD values for redocking validation of all protein targets.

Protein	PDB ID	Crystallized Ligand in Redocking PDB	Binding Pocket	Average RMSD (Å)	Upper Bound (Å)	Lower Bound (Å)	Interpretation	Interactions
AKT1	3O96	Flibanserin	Same allosteric pocket	2.066	5.967	0.563	Acceptable	Hydrophobic: TRP 80, VAL 270, ILE 84, LEU 264, LEU 210, ASP 292 Hydrogen bonds: SER 205, HOH 455 Electrostatic: LYS 268, ARG 273
MAPK3	4QTB	Camonsertib	Same kinase active site	4.382	10.673	1.285	Higher RMSD	Hydrophobic: ALA 52, TYR 81, ARG 84, ILE 73, LEU 173, VAL 56, ALA 69, MET 125 Hydrogen bonds: LYS 131, TYR 53, ASP 123, ASP 128 Electrostatic: ASP 184 Other: THR 85
CDK6	5L2T	Ribociclib	Same ATP-binding pocket	0.378	1.152	0.076	Acceptable	Hydrophobic: VAL 77, ALA 41, LEU 152, VAL 101, ILE 19, PHE 98 Hydrogen bonds: GLU 99, ASP 163, ASP 104 Electrostatic: HIS 100 Other: LYS 43
JAK1	6N7A	JAK1 inhibitor	Same catalytic pocket	1.482	4.174	0.226	Acceptable	Hydrophobic: LEU 959, LEU 881, LEU 1010, VAL 889, ALA 906 Hydrogen bonds: GLU 957, GLY 962, GLY 1020 Electrostatic: ARG 1007 Other: ASN 1008
JAK2	3E64	Ruxolitinib	Same ATP pocket	1.448	3.34	0.517	Acceptable	Hydrophobic: VAL 863, ALA 880, LEU 855, LEU 983 Hydrogen bonds: GLU 930, LEU 932 Electrostatic: MET 929
SRC	7NG7	PD082514	Same SRC active site	1.944	6.023	0.671	Acceptable	Hydrophobic: VAL 284, LEU 396, ALA 296, ALA 406, PHE 408, GLY 347, MET 344 Hydrogen bonds: THR 341, GLU 342, ASP 407 Electrostatic: LYS 298 Other: ILE 339
CTNNB1	7ZRB	RS6452	Same Armadillo repeat domain (alternate PDB ligand shifted 100–200 aa)	3.8	8.908	1.615	Higher RMSD	Hydrophobic: CYS 429, LYS 508 Other (proximity): ASN 426, ASP 390
STAT3	6NUQ	Fruquintinib	Same SH2 domain	2.735	7.103	0.55	High RMSD	Hydrophobic: VAL 637, PRO 639, ILE 659, TYR 657 Hydrogen bonds: SER 611, SER 613, SER 614, SER 636, GLU 612, GLU 638, GLN 644, TYR 640, TRP 623 Electrostatic: ARG 609 Other: MET 660
CDK4	9CSK	Atirmociclib	Same ATP pocket	0.332	0.813	0.081	Acceptable	Hydrophobic: VAL 20, VAL 96, ILE 12, LEU 147, ALA 33, TYR 17 Hydrogen bonds: GLU 94, HIS 95, ASP 158 Electrostatic: LYS 35
PIK3CA	4JPS	Alpelisib	Same ATP-binding pocket	0.619	2.522	0.049	Acceptable	Hydrophobic: VAL 850, VAL 772, ILE 932, ILE 848, ILE 800, LYS 802, TYR 836 Hydrogen bonds: GLN 859, VAL 851, SER 854, HIS 855 Electrostatic: MET 922

Among the ten proteins studied, CDK4, CDK6, PIK3CA, JAK1, JAK2, and SRC demonstrated excellent reproducibility, with RMSD values below the accepted threshold of ≤2.0 Å, confirming the reliability of the docking parameters for rigid or well-defined binding pockets. CDK4 (0.332 Å), CDK6 (0.378 Å), and PIK3CA (0.619 Å) showed near-exact reproduction of their crystallographic poses, indicating high methodological precision. AKT1 (2.066 Å), although marginally above the threshold, remained acceptable and is consistent with prior reports describing substantial conformational flexibility within its allosteric pocket [33]. STAT3 exhibited a moderate deviation (2.735 Å), aligning with literature that identifies its SH2 binding interface as structurally plastic and challenging to redock with low RMSD values [34]. Higher RMSDs for CTNNB1 (3.800 Å) and MAPK3 (4.382 Å) were similarly anticipated. β-catenin’s armadillo repeat domain is known to undergo significant conformational fluctuations [35], while MAPK3’s activation loop exists in multiple dynamic states, making accurate pose reproduction inherently difficult [36].

Overall, despite expected deviations in targets with flexible binding regions, the majority of proteins demonstrated strong crystallographic pose recovery, confirming that the docking workflow is robust, reproducible, and suitable for reliable structure–based analysis of Vernolac’s phytochemicals.

### Molecular dynamics (MD) simulations

**Fig 14:**
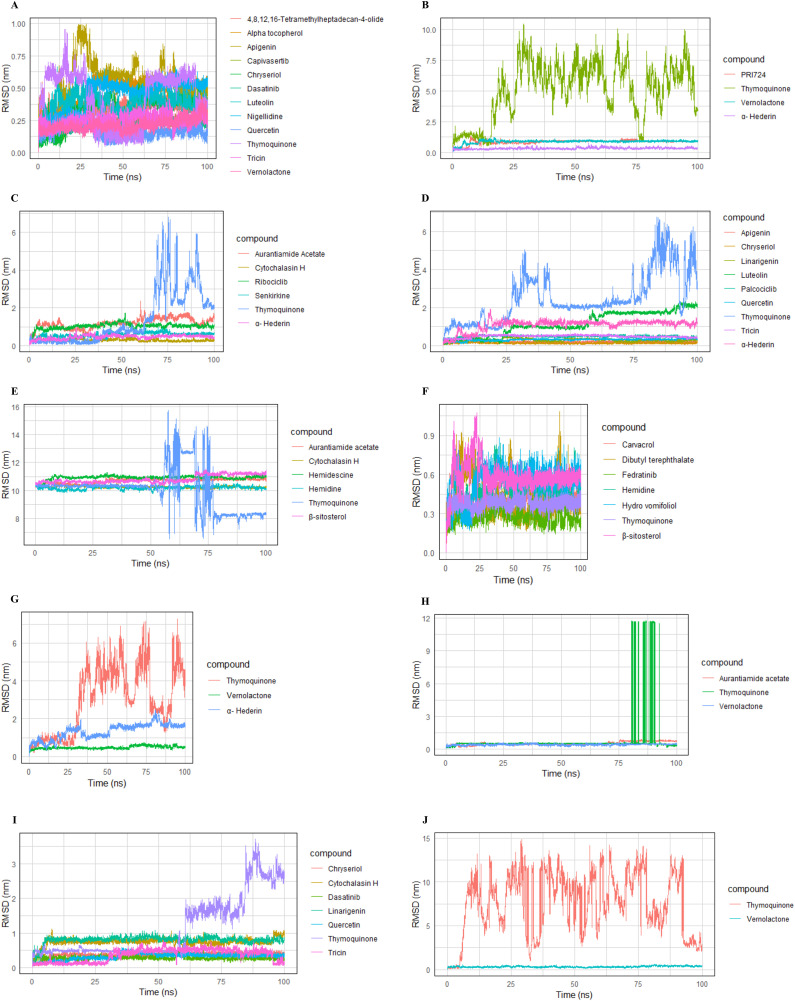
The analysis of the Root Mean Square Deviation (RMSD) of the target proteins and their respective compounds. **A**: AKT1 complex, **B**: CTNNB1 complex, **C**: CDK4 complex, **D**: CDK6 complex, **E**: JAK1 complex, **F**: JAK2 complex, **G**: MAPK3 complex, **H**: PIK3CA complex, **I**: SRC complex, **J**: STAT3 complex.

**Fig 15:**
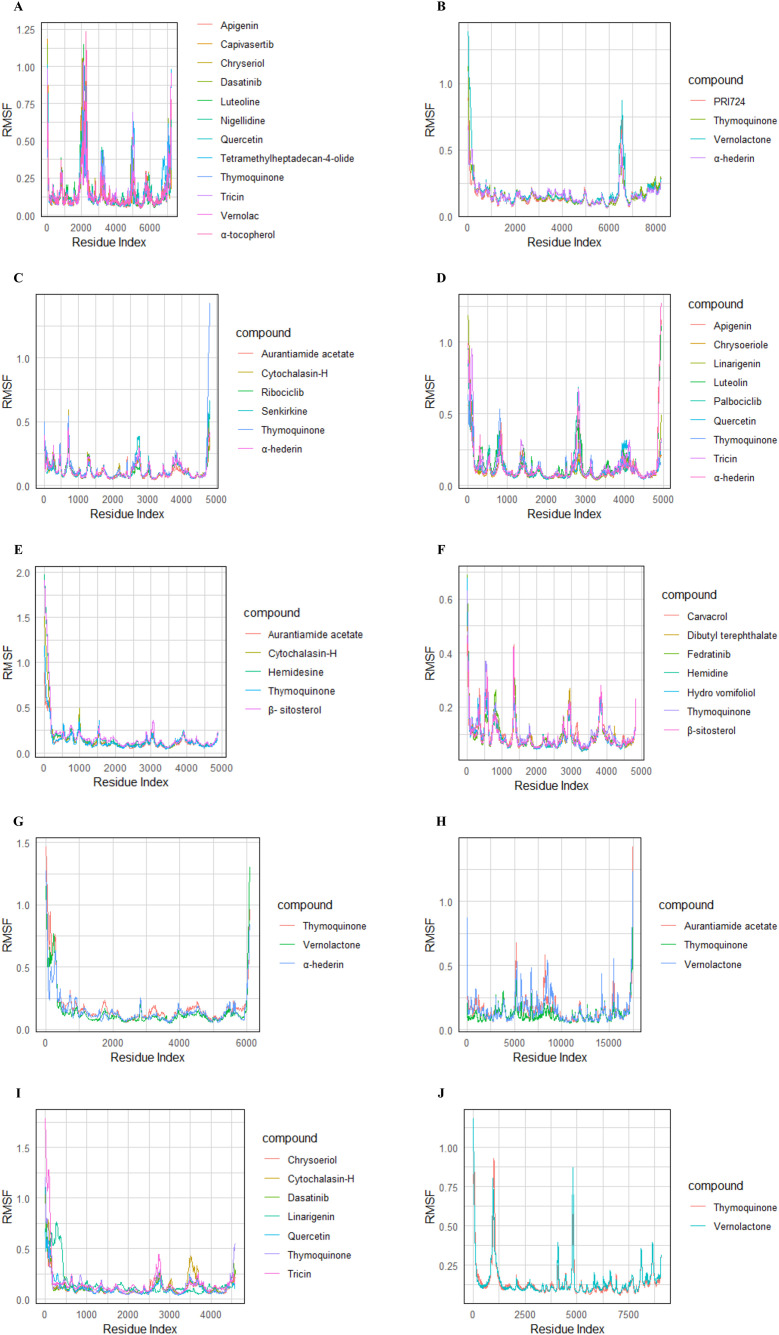
The analysis of the Root Mean Square Fluctuation (RMSF) of the target proteins and their respective compounds. **A**: AKT1 complex, **B**: CTNNB1 complex, **C**: CDK4 complex, **D**: CDK6 complex, **E**: JAK1 complex, **F**: JAK2 complex, **G**: MAPK3 complex, **H**: PIK3CA complex, **I**: SRC complex, **J**: STAT3 complex.

The docking results were verified using a 100 ns MD simulation, providing significant structural insights such as conformational changes and stability of the protein-ligand complex. The RMSD (Fig 14) and RMSF (Fig 15) analyses provided insights into structural dynamics. Fig 14 depicts the RMSD plots for the ten target proteins, AKT1, CTNNB1, PIK3CA, STAT3, MAPK3, CDK4, CDK6, JAK1, JAK2, and SRC, and their respective compounds, and RMSD values are presented in Table 8. A (0.1–0.2) nm deviation range between the RMSD of the ligand in the complex is considered acceptable and stable, while deviations reaching up to (0.3–0.4) nm are still acceptable for some compounds, depending on the presence of specific flexible regions. The evaluation is based on the mean RMSD, where a lower value signifies a more stable and rigid binding pose, while a higher value indicates greater conformational flexibility. The standard deviation further quantifies the extent of fluctuations in each compound’s binding.

**Table 8:** Summary of the mean Root Mean Square Deviation (RMSD) for natural compounds docked to the AKT1, β-catenin, CDK4, CDK6, JAK1, JAK2, MAPK3, STAT3, PIK3CA, and SRC proteins.

Target protein	Compound	Mean RMSD (nm)
AKT1	Quercetin	0.17 ± 0.05
	Dasatinib (reference)	0.21 ± 0.04
	Vernolactone	0.21 ± 0.05
	Capivasertib (reference)	0.24 ± 0.03
	Tricin	0.25 ± 0.13
	Chryseriol	0.33 ± 0.10
	4,8,12,16-Tetramethylheptadecan-4-olide	0.35 ± 0.05
	Luteolin	0.38 ± 0.07
	Thymoquinone	0.43 ± 0.20
	α-tocopherol	0.47 ± 0.11
	Nigellidine	0.50 ± 0.05
	Apigenin	0.53 ± 0.18
CTNNB1	PRI724 (reference)	0.86 ± 0.17
	Vernolactone	0.88 ± 0.13
	α-Hederin	0.33 ± 0.07
	Thymoquinone	5.08 ± 2.29
CDK4	Ribociclib (reference)	1.02 ± 0.17
	α-Hederin	0.46 ± 0.10
	Cytochalasin H	0.29 ± 0.07
	Senkirkine	0.57 ± 0.12
	Thymoquinone	1.36 ± 1.41
	Aurantiamide Acetate	1.18 ± 0.27
CDK6	Palcociclib (reference)	0.48 ± 0.05
	Chryseriol	0.13 ± 0.03
	Quercetin	0.31 ± 0.05
	Apigenin	0.20 ± 0.03
	α-Hederin	1.08 ± 0.29
	Tricin	0.49 ± 0.07
	Thymoquinone	2.49 ± 1.29
	Luteolin	1.15 ± 0.62
	Linarigenin	0.30 ± 0.07
JAK1	Cytochalasin H	10.26 ± 0.07
	Aurantiamide acetate	10.69 ± 0.16
	Hemidescine	10.92 ± 0.14
	β-sitosterol	10.81 ± 0.27
	Hemidine	10.19 ± 0.11
	Thymoquinone	10.10 ± 1.48
JAK2	Fedratinib (reference)	0.28 ± 0.06
	Carvacrol	0.42 ± 0.06
	Dibutyl terephthalate	0.43 ± 0.15
	Thymoquinone	0.38 ± 0.04
	Hemidine	0.48 ± 0.10
	Hydro vomifoliol	0.55 ± 0.12
	β-sitosterol	0.58 ± 0.11
MAPK3	α-Hederin	1.34 ± 0.40
	Vernolactone	0.48 ± 0.07
	Thymoquinone	2.99 ± 1.75
STAT3	Vernolactone	0.34 ± 0.08
	Thymoquinone	7.69 ± 3.54
PIK3CA	Vernolactone	0.40 ± 0.07
	Aurantiamide acetate	0.50 ± 0.16
	Thymoquinone	0.79 ± 1.84
SRC	Dasatinib (reference)	0.28 ± 0.05
	Linarigenin	0.80 ± 0.11
	Chryseriol	0.37 ± 0.07
	Cytochalasin H	0.74 ± 0.12
	Quercetin	0.32 ± 0.09
	Tricin	0.36 ± 0.19
	Thymoquinone	1.11 ± 0.87

For the AKT1 target protein, a broad spectrum of stability profiles was observed. Quercetin (0.17 ± 0.05 nm) stands out as the most stable binder, with the lowest mean RMSD. This low deviation signifies a highly stable binding and minimal conformational fluctuations within the active site. The reference compounds, Dasatinib (0.21 ± 0.04 nm) and Capivasertib (0.24 ± 0.03 nm), and the compound Vernolactone (0.21 ± 0.05 nm) also demonstrate high stability. Tricin (0.25 ± 0.13 nm), Chryseriol (0.33 ± 0.10 nm), 4,8,12,16-Tetramethylheptadecan-4-olide (0.35 ± 0.05 nm), and Luteolin (0.38 ± 0.07 nm) represent moderately stable binding, showing mean RMSD values in the middle range. Nigellidine (0.50 ± 0.05 nm), and Apigenin (0.53 ± 0.18 nm) exhibited higher mean RMSD values. These elevated values indicate a more flexible and dynamic interaction network with the AKT1 target, with Nigellidine successfully maintaining a steady, well-accommodated trajectory after its initial equilibration phase.

In the β-catenin system, α-Hederin (0.33 ± 0.07 nm) is the most stable compound, with the lowest mean RMSD, indicating a highly consistent binding pose. The reference control, PRI724 (0.86 ± 0.17 nm), and Vernolactone (0.88 ± 0.13 nm) shows high stability despite slightly elevated RMSD values. In contrast, Thymoquinone (5.08 ± 2.29 nm) is the least stable, with the highest mean RMSD and a very large standard deviation, suggesting significant movement and flexibility within the binding site.

For the CDK4 protein, Cytochalasin H (0.29 ± 0.07 nm) is the most stable compound, maintaining a highly consistent and rigid binding pose. α-Hederin (0.46 ± 0.10 nm) and Senkirkine (0.57 ± 0.12 nm) show moderate stability. The reference compound, Ribociclib (1.02 ± 0.17 nm), along with Aurantiamide Acetate (1.18 ± 0.27 nm), is considerably less stable. Thymoquinone (1.36 ± 1.41 nm) is the least stable, with the highest mean RMSD and the largest standard deviation, suggesting significant movement and flexibility.

Within the CDK6 system, Chryseriol (0.13 ± 0.03 nm) is the most stable compound, indicating it maintains a highly consistent and rigid binding pose. Apigenin (0.20 ± 0.03 nm), Quercetin (0.31 ± 0.05 nm), and Linarigenin (0.30 ± 0.07 nm) also demonstrate high stability. The reference control, Palcociclib (0.48 ± 0.05 nm), and Tricin (0.49 ± 0.07 nm) show high RMSD values. However, they maintained a steady, well-accommodated trajectory after the initial equilibration phase. α-Hederin (1.08 ± 0.29 nm) and Luteolin (1.15 ± 0.62 nm) consist of notably high RMSDs yet stable trajectories, while Thymoquinone (2.49 ± 1.29 nm) is the least stable with frequent fluctuations and the highest mean RMSD.

For the JAK1 system, a unique stability profile is observed. Thymoquinone (10.10 ± 1.48 nm) has a comparatively low mean RMSD, despite its high standard deviation, suggesting significant conformational fluctuations. In contrast, Hemidescine (10.92 ± 0.14 nm) has the highest mean RMSD. However, a steady, well-accommodated trajectory was observed. Other compounds, including Hemidine (10.19 ± 0.11 nm), Cytochalasin H (10.26 ± 0.07 nm), Aurantiamide acetate (10.69 ± 0.16 nm), and β-sitosterol (10.81 ± 0.27 nm), fall between these two extremes whereas all of these compounds show steady trajectories with less fluctuations.

Analyzing the stability of the JAK2 protein, Fedratinib (0.28 ± 0.06 nm) serves as the reference compound and demonstrates the highest stability. All other compounds, including Carvacrol (0.42 ± 0.06 nm), Dibutyl terephthalate (0.43 ± 0.15 nm), Thymoquinone (0.38 ± 0.04 nm), Hemidine (0.48 ± 0.10 nm), Hydro vomifoliol (0.55 ± 0.12 nm) and β-sitosterol (0.58 ± 0.11 nm), exhibit higher mean RMSD values. However, moderate stability was observed throughout the trajectory with minimal fluctuations.

The analysis of the MAPK3 system reveals Vernolactone (0.48 ± 0.07 nm) maintains a lower mean RMSD, suggesting that it is the most stable compound, maintaining a structural conformational stability. Meanwhile, Thymoquinone has a mean RMSD of 2.99 ± 1.75 nm. This value suggests a significant degree of conformational movement, indicating that the compound is not highly stable in its binding to the MAPK3 protein.

For the STAT3 protein, Vernolactone (0.34 ± 0.08 nm) is the more stable compound, as indicated by its lower mean RMSD, suggesting that it maintains a more consistent and rigid binding pose. Thymoquinone (7.69 ± 3.54 nm) is the least stable, with a significantly higher mean RMSD and a large standard deviation, suggesting substantial movement and flexibility.

In the PIK3CA system, Vernolactone (0.40 ± 0.07 nm) is the most stable compound, indicating it maintains a more consistent and rigid binding pose. Aurantiamide acetate (0.50 ± 0.16 nm) is moderately stable. In contrast, Thymoquinone (0.79 ± 1.84 nm) is the least stable with the highest mean RMSD and a large standard deviation, suggesting significant movement and flexibility within the binding site.

For the SRC protein, Dasatinib (0.28 ± 0.05 nm) serves as the reference compound and is the most stable compound. The other compounds are all less stable, as evidenced by their higher mean RMSD values. Quercetin (0.32 ± 0.09 nm), Tricin (0.36 ± 0.19 nm), and Chryseriol (0.37 ± 0.07 nm) are relatively stable binders. In contrast, Cytochalasin H (0.74 ± 0.12 nm), Linarigenin (0.80 ± 0.11 nm), and especially Thymoquinone (1.11 ± 0.87 nm) are considerably less stable, with Thymoquinone having the highest mean RMSD and a very high standard deviation, indicating the most flexible binding pose.

RMSF is a key metric in molecular dynamics simulations that measures the average deviation of an atom or residue from its reference position, providing insight into the flexibility of a protein-ligand complex. RMSF analysis was employed to evaluate the flexibility of amino acid residues and ligand atoms during the simulation [33,37]. A lower RMSF value indicates a more rigid and stable complex, suggesting a stronger binding interaction, while a higher RMSF points to a more flexible and less stable complex.

The RMSF values for protein-ligand complexes are summarized in Table 9. Across the provided datasets, the RMSF analysis reveals a range of stability among the protein-ligand pairs. For instance, the JAK2 protein demonstrates the most stable interactions overall, with its ligands having the lowest mean RMSF values in the dataset. Dibutyl terephthalate and Hemidine stand out with exceptionally low RMSF values of (0.090 ± 0.054) nm and (0.090 ± 0.058) nm, respectively, indicating highly rigid complexes. In contrast, the most flexible interactions were observed for JAK1 with β-sitosterol (0.195 ± 0.255 nm) and MAPK3 with Thymoquinone (0.192 ± 0.188 nm). The large standard deviations for these compounds suggest significant fluctuations and less-defined binding poses. The data for AKT1, CTNNB1, CDK4, CDK6, PIK3CA, and SRC fall within this range, each showing a clear hierarchy from most stable to least stable ligand based on their mean RMSF values.

**Table 9:** Summary of the mean Root Mean Square Fluctuation (RMSF) for natural compounds docked to the AKT1, β-catenin, CDK4, CDK6, JAK1, JAK2, MAPK3, STAT3, PIK3CA, and SRC proteins.

Target protein	Compound	Mean RMSF (nm)
**AKT1**	Thymoquinone	0.15 ± 0.11
	Capivasertib	0.15 ± 0.15
	Vernolac	0.15 ± 0.12
	Dasatinib	0.16 ± 0.14
	Luteoline	0.16 ± 0.16
	Nigellidine	0.16 ± 0.11
	Apigenin	0.16 ± 0.17
	Quercetin	0.16 ± 0.16
	α-tocopherol	0.16 ± 0.16
	Tetramethylheptadecan-4-olide	0.17 ± 0.15
	Chryseriol	0.17 ± 0.16
	Tricin	0.17 ± 0.15
**CTNNB1**	PRI724	0.15 ± 0.09
	Thymoquinone	0.16 ± 0.09
	α-hederin	0.16 ± 0.07
	Vernolactone	0.18 ± 0.14
**CDK4**	Aurantiamide acetate	0.11 ± 0.07
	Ribociclib	0.12 ± 0.07
	Cytochalasin-H	0.12 ± 0.07
	α-hederin	0.12 ± 0.07
	Senkirkine	0.12 ± 0.09
	Thymoquinone	0.14 ± 0.14
**CDK6**	Chrysoeriole	0.11 ± 0.08
	Linarigenin	0.11 ± 0.12
	Palbociclib	0.12 ± 0.11
	Thymoquinone	0.12 ± 0.09
	α-hederin	0.13 ± 0.14
	Quercetin	0.13 ± 0.12
	Apigenin	0.13 ± 0.14
	Tricin	0.14 ± 0.13
	Luteolin	0.15v± 0.13
**JAK1**	Aurantiamide acetate	0.14 ± 0.11
	Thymoquinone	0.14 ± 0.14
	Cytochalasin-H	0.16 ± 0.19
	Hemidesine	0.18 ± 0.26
	β- sitosterol	0.20 ± 0.26
**JAK2**	Dibutyl terephthalate	0.09 ± 0.05
	Hemidine	0.09 ± 0.06
	Hydro vomifoliol	0.09 ± 0.06
	Fedratinib	0.09 ± 0.06
	Thymoquinone	0.10 ± 0.06
	Carvacrol	0.10 ± 0.06
	β- sitosterol	0.10 ± 0.06
**MAPK3**	Vernolactone	0.15 ± 0.16
	α-hederin	0.160 ± 0.14
	Thymoquinone	0.19 ± 0.19
**PIK3CA**	Thymoquinone	0.12 ± 0.07
	Aurantiamide acetate	0.15 ± 0.10
	Vernolactone	0.15 ± 0.12
**SRC**	Chrysoeriol	0.11b± 0.10
	Quercetin	0.17 ± 0.09
	Dasatinib	0.12 ± 0.10
	Cytochalasin-H	0.13 ± 0.13
	Thymoquinone	0.13 ± 0.13
	Linarigenin	0.15 ± 0.16
	Tricin	0.16 ± 0.21

To understand how these compounds fit into the active site and influence its structure, the fluctuation of individual amino acid residues directly onto the functional regions of the proteins was studied. While considering a broad range of protein averages, distinct residues were studied more closely.

Considering the AKT1-Nigellidine complex, the residue LEU: A:210 positioned deep inside the activation site (residues 201−216), showed outstanding positional constraint, maintaining a restricted fluctuation between 0.1024 nm and 0.1022 nm. This aligns with the fact that a hydrophobic bond was formed as shown in Fig 12-1K. Simultaneously, another residue site, TYR: A:272, deep inside the active site, along with residue sites VAL: A:270, LYS: A:268 and LEU: A:264 showed fluctuations between 0.1385 nm – 0.1327 nm, indicating moderate flexibility of the active site. Moving toward the mouth of the binding channel, the residue TRP: A:80 registered fluctuations between 0.1889 nm and 0.1822 nm, indicating moderate flexibility.

Within the AKT1-Quercetin complex, the active site pocket exhibited profound structural containment. The active sites near the mouth of the pocket, ASN:A:54 and GLN:A:79 maintained exceptionally low fluctuations ranging at 0.3907 nm and 0.1931 nm respectively. deep within the pocket, residues LEU:A:264, and VAL:A:270 demonstrated rigid positional constraints with RMSF tightly confined between 0.1015 nm and 0.1020 nm. Furthermore, residue LEU:A:210 present in the core activation loop [38] demonstrated minimal fluctuation within a highly restricted area of 0.1005 nm to 0.1020 nm (residues 201–216).

The tracking of residues within AKT1–Tetramethylheptadecan-4-olide complex showed distinct stabilization patterns across the domains.

Similar to AKT1-Quercetin complex, deep within the pocket, residues LEU:A:264, LYS:A:268 and VAL:A:270 showed structural constraint with fluctuations between 0.0815 nm and 0.0742 nm. Concurrently, residue LEU:A:210 at the core activation loop demonstrated a highly locked position that fluctuated within a narrow area of 0.0899 nm to 0.0848 nm (residues 201–216). Moving outward towards the entry of the mouth of the pocket, residue TRP:A:80 showed slightly elevated RMSF (0.1986 nm to 0.1757 nm) values, indicating moderate flexibility.

To understand how Vernolactone structurally works on the active site of β-catenin (CTNNB1), the fluctuation behaviors of the active site pocket residues were studied. The data reveals that specific residues coordinating the compound maintain exceptional structural containment, staying below traditional 0.25 nm fluctuation threshold. The hydrogen bonding residues, ARG: A:469 and GLY: A:512 demonstrated profound local rigidity, registering tight fluctuations of 0.2472 nm and 0.2071 nm, respectively. The neighboring pocket boundaries at residues 471 and 484 mirrored this stability, tracking closely at 0.2388 nm and 0.2341 nm. Similarly, the residues 486, 488, 500, and 502 displayed highly managed, low-to-moderate mobility behaviors, with absolute values tightly restricted between 0.2447 nm and 0.2312 nm. Most notably, the residue at the opening of the active site pocket, LYS: A:508 achieved a stable localized fluctuation around 0.2234 nm, indicating that the entire pocket undergoes profound structural rigidification upon ligand binding.

For the CDK4-Cytochalasin H complex, the critical activation loop anchor residue [39] ARG:A:186 exhibited rigidity with a fluctuation between 0.1327 nm and 0.1246 nm. Meanwhile, the CDK4–α-Hederin’s loop gate residue ALA:A:167 and neighboring loop segments exhibited 0.1616 nm to 0.148 nm range fluctuation indicating moderate flexibility.

The residue mapping of the JAK2–Thymoquinone complex revealed that different zones in the protein were induced with rigidity and flexibility. The primary hinge-region loop [40] gateway residue LEU:A:932, alongside residue TYR: A:931 achieved highly stable trajectories with fluctuations between 0.0968 nm and 0.0911 nm (928–939 residues). Deep within the pocket, residues VAL:A:863, ALA:A:880, and LEU:A:983 showed a remarkable suppressed fluctuation dropping from 0.1372 nm to 0.0686 nm (862–984 residues).

Similarly, the JAK2–Carvacrol complex demonstrated the central anchor residue LEU:A:932 maintained an exceptionally stable trajectory, with its atomic fluctuations strictly bound between 0.1115 nm and 0.1095 nm (928–939 residues). Deep within the active site pocket residues VAL:A:863, ALA:A:880, and LEU:A:983 exhibited a heavily suppressed fluctuations between 0.1466 nm and 0.1115 nm indicating moderate flexibility (862–984 residues).

### GC-MS analysis

The GC-MS of the supercritical extract of Vernolac identified multiple phytochemical constituents, including α-tocopherol acetate, α-pinene, β-myrcene, β-pinene, D-limonene, dodecanoic acid, carvacrol, cycloartenol, thymoquinone, 1,3,5-trimethyl benzene, α-terpinyl acetate, hexadecenoic acid, linalyl acetate, stigmasterol, octadecanoic acid, oleic acid, palmitic acid, and lauric acid. The complete list of phytochemicals identified by GC-MS is provided in S5 Table.

### *In vitro* antiproliferative activity

**Fig 16:**
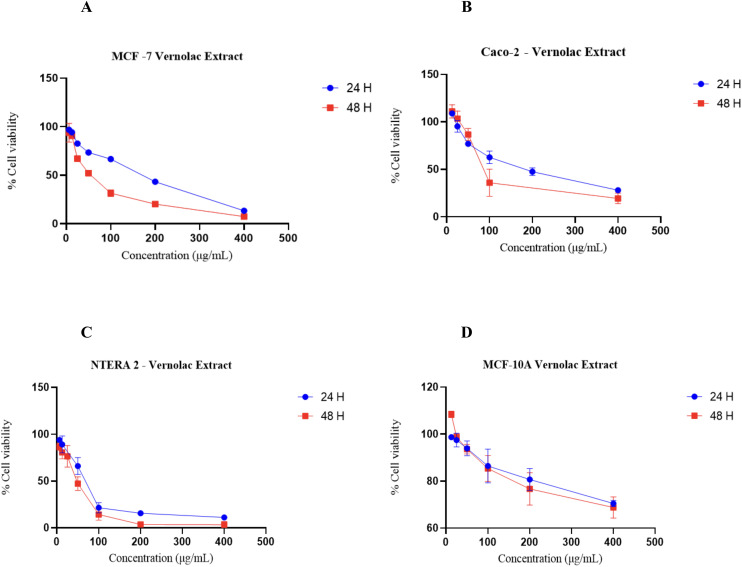
Cell viability (%) of MCF-7, Caco-2, NTERA-2 cl.D1, and MCF-10A cells following treatment with Vernolac, assessed by SRB assay at 24 h and 48 h post-incubation. **A**: MCF-7, **B**: Caco-2, **C**: NTERA-2 cl.D1, **D**: MCF-10A.

The *in vitro* antiproliferative effects of Vernolac on three cancer cell lines (MCF-7, Caco-2, and NTERA-2 cl.D1) and a normal cell line (MCF-10A) were evaluated using the SRB assay following 24 h and 48 h post-incubation. As shown in Table 10, a dose-dependent reduction in cell viability was observed across the tested cancer cell lines. The IC_50_ values demonstrated increased antiproliferative activity with prolonged exposure (Fig 16). Importantly, the non-tumorigenic MCF-10A cells demonstrated higher IC_50_ values at 24 h and 48 h, suggesting a considerably lower antiproliferative effect of Vernolac on normal cells compared to cancer cells. These findings suggest a potential selective antiproliferative effect of Vernolac toward cancer cells.

**Table 10:** The IC_50_ values (µg/mL) of Vernolac in MCF-7, Caco-2, NTERA 2, and MCF-10A cells at 24 h and 48 h incubation periods, as determined by SRB assay.

Cell line	IC_50_ (24 h) (μg/mL)	IC_50_ (48 h) (μg/mL)
MCF-7	124.5 ± 1.46	54.01 ± 1.02
CaCo-2	173.2 ± 2.32	85.52 ± 1.63
NTERA-2	61.5 ± 0.74	42.41 ± 1.06
MCF-10A	1075 ± 1.69	803.5 ± 1.51

## Discussion

Network pharmacology is an emerging paradigm in drug discovery and development, offering a systematic approach to explore complex interactions between compounds, molecular targets, and disease pathways [9,10]. In contrast to the conventional “one drug-one target” model, network pharmacology acknowledges the multifactorial nature of diseases such as cancer. This is particularly beneficial for investigating the mechanisms of action of polyherbal formulations that act through multiple targets and pathways simultaneously. The present study employed a network pharmacology-based approach to explore the potential mechanisms of action of Vernolac, a polyherbal nutraceutical composed of five traditionally used medicinal plants in Sri Lanka. The formula-herb-compound-target-disease network, cluster analysis of the core PPI network, hub node analysis, and topology analysis revealed a set of key hub proteins, including AKT1, EGFR, STAT3, CTNNB1, BCL2, JUN, SRC, MAPK3, and JAK1. These proteins are commonly dysregulated in various types of cancer [41,42]. KEGG pathway enrichment analysis of Vernolac revealed that these targets are significantly enriched in several pathways associated with prostate cancer, glioma, non-small lung carcinoma (NSCLC), endometrial cancer, bladder cancer, pancreatic cancer, renal cell carcinoma, and myeloid leukemias (acute and chronic myeloid leukemias). Results of the present study highlight the broad-spectrum potential of Vernolac against multiple cancer types. Literature reports that several major phytochemicals found in Vernolac have been previously experimentally validated for their modulatory effects on these hub proteins [17,18,22].

The virtual ligand library employed in this study represents a curated phytochemical space derived from reported constituents of the medicinal plants present in Vernolac, encompassing both major and minor compounds. The GC-MS profile was primarily generated as a chemical fingerprint of the extract rather than a comprehensive representation of all bioactive constituents. As GC-MS analysis detects compounds, primarily limited to volatile and thermally stable constituents [43]. Therefore, the discrepancies between docking hits (e.g., nigellidine) and GC-MS results may arise due to analytical limitations, low compound concentrations in the sample or physicochemical properties restricting detectability. Importantly, the absence or low abundance of a compound in GC-MS does not preclude its biological relevance, as pharmacologically active phytochemicals often occur in trace amounts. This highlights a partial but not complete overlap between the virtual and experimental chemical spaces.

Vernolactone, a sesquiterpene lactone isolated from *V. zeylanica*, mediated significant cytotoxic effects in MDA-MB-231 breast cancer cells, with minimal effects on normal mammary epithelial cells (MCF-10A). Moreover, this study reports that Vernolactone exerts pro-apoptotic effects by upregulating *p53* and *Bax*, downregulating *survivin* and Heat Shock Protein (HSP) complex-related genes [44]. Abeysinghe et al. report that vernolactone mediates its antiproliferative activity via apoptosis and autophagy in cancer stem-like cells (NTERA-2), with minimal antiproliferative effects in noncancerous peripheral blood mononuclear cells (PBMC) [18]. As evident from the gene expression results of the same study, vernolactone significantly downregulates the expression of PI3K, AKT, and mTOR, demonstrating its potential to target CSCs, inhibiting the PI3K/Akt/mTOR pathway [18]. Molecular docking and dynamics simulation analyses of the present study identified β-catenin (CTNNB1) as a novel target of vernolactone. To our knowledge, this is the first report to suggest a direct interaction between vernolactone and β-catenin, a key player in the Wnt/β-catenin signaling pathway. Vernolactone exhibited outstanding stability in molecular dynamics, maintaining a stable and steady trajectory throughout 100 ns. Furthermore, RMSF results show that by forming tight hydrogen bonds with core residues (ARG:A:469 and GLY:A:512), the activity of β-catenin is heavily suppressed, providing a clear mechanism for its downregulation.

This novel finding broadens the mechanistic understanding of the anticancer potential of vernolactone, particularly targeting CSCs.

Thymoquinone, a phytochemical found in *N. sativa*, exhibits anticancer and chemopreventive effects across a broad range of cancer types, targeting multiple molecular pathways [45]. According to previous *in vitro* and *in vivo* studies, its mechanism of action mainly involves the induction of apoptosis via reactive oxygen species (ROS) generation [46], modulating pro- and anti-apoptotic proteins such as Bax, Bcl-2, and caspases, and inhibiting key signaling pathways including PI3K/Akt, and JAK/STAT [45]. In the present study, thymoquinone demonstrated exceptional stability during molecular dynamics, with the target protein JAK2 maintaining a mean RMSD value of 0.38 ± 0.04 nm with minimal fluctuations. Furthermore, RMSF analysis shows that the clamping of the hinge region (LEU:A:932) and rigidity in the pocket (VAL:A:863, ALA:A:880, and LEU:A:983). The rigidity of these zones suggests freezing of the domain, effectively leading to the downregulation of the JAK2 target protein [40].

Carvacrol, a monoterpenoid phenol abundant in *N. sativa* [47], exhibits broad-spectrum anticancer effects by modulating multiple signaling pathways that align closely with the signaling pathways and hub nodes identified in the present study. According to the literature, its anticancer mechanisms involve the modulation of Bax, Bcl-2, cytochrome c, CDK4/6, p53, caspases (3/6/7/8/9), and cyclins (A/B/D1) [47,48]. Carvacrol also suppresses key signaling pathways, including PI3K/AKT, MAPKs (p-ERK, p-JNK, p-38), and ROS generation [49–52]. Previous studies conducted on breast cancer cells (MDA-MB-231 and MCF-7) demonstrate that carvacrol mediates cell growth inhibition and apoptosis by upregulating Bax and PI3K/p-AKT and TRPM7 suppression [50,53,54]. Similarly, a plethora of *in vitro* cancer cell models including cervical cancer (HeLa cells), choriocarcinoma (JAR and JEG3 cells), lung cancer (A549 and H460), colorectal cancer (Caco-2, HT-29, LoVo cells), prostate cancer (PC-3 cells), melanoma (A375 cells), gastric adenocarcinoma (AGS cells), glioblastoma (U87 cells), and liver cancer (HepG2 cells) have been tested with carvacrol and has strongly validated its anticancer potential [49,51,55–57]. Importantly, carvacrol downregulates STAT3, JAK2 and MMP2 [51,52], three important targets identified by the present network pharmacology study. These findings are further supported by *in vivo* studies demonstrating carvacrol’s ability to suppress tumor growth [55,56], highlighting its potential contribution to the multi-targeted anticancer effects of Vernolac. In the present study, molecular dynamics simulations of carvacrol with JAK2 revealed steady trajectories with a mean RMSD of 0.42 ± 0.06 nm. Similar to thymoquinone, RMSF analysis strongly suggests that the freezing of the domain effectively leads to the downregulation of the JAK2 target protein.

Another prominent phytochemical present in Vernolac is α-hederin, a triterpenoid found in *N. sativa*. Previous studies report that α-hederin induces ROS-dependent activation of AMPK/mTOR signaling pathway in colorectal cancer cells (HCT116 and HCT8), leading to both apoptosis and autophagy [58]. It also disrupts mitochondrial membrane potential, elevates Bax/Bcl-2 ratio, activates caspases (3/9) via ROS-mediated mitochondrial apoptosis in gastric (HGC27/DPP), and breast cancer (MCF-7 and MDA-MB-231) models [59,60]. Moreover, α-hederin reduces p-AKT, p-PI3K, and p-mTOR levels in oral cancer cells (SCC-25), thereby suppressing the PI3K/Akt/mTOR pathway [61]. According to literature, α-hederin exhibits a broad-spectrum anticancer potential against numerous *in vitro* and *in vivo* cancer models, including ovarian, lung, colorectal, esophageal, and liver cancer [62]. A recent study reports that α-hederin is a potent inhibitor of Wnt/β-catenin pathway genes (cyclin D1 and CD44), and induces apoptosis in breast CSCs [63]. Cyclin D1 binds with CDK4 and CDK6, forming an active complex that promotes cell cycle progression [64]. Therefore, inhibition of cyclin D1 may reduce CDK4 activity by limiting the formation of the cyclin D1-CDK4 complex. However, to the best of our knowledge, no prior studies have reported a direct interaction between α-hederin and CDK4.

Findings of the present study suggest that α-hederin directly targets CDK4 establishing a highly stable complex without causing ligand unbinding. RMSD results suggest a steady stable trajectory, while RMSF analysis highlights an induced-fit mechanism driven by steric hindrance. The core of α-hederin forms hydrophobic interactions that pin down deep pocket residues such as ALA: A:21, LEU: A:64, meanwhile the activation loop’s residue also forms a flexible hydrophobic interaction at ALA: A:167. This highlights a novel mechanism of action that may contribute to its anticancer effects, independent of its modulation of Cyclin D1.

Quercetin is a flavonoid abundant in *H. indicus* and *S. glabra* with well-established anticancer mechanisms through numerous signaling pathways, including Wnt/β-catenin, MAPK/ERK1/2, p53, JAK/STAT, AMPKα/ASK1/p38, RAGE/PI3K/AKT/mTOR axis, and NF-κB [65]. As evident in the literature, quercetin exhibits ROS-mediated anticancer activity against CSCs [66]. Quercetin also alters Numbl and Notch levels in pancreatic ductal adenocarcinoma cells, inhibiting the self-renewal and aggressiveness of CSCs [67]. According to Seo et al., quercetin induces caspase-dependent extrinsic apoptosis in HER2-overexpressing BT-474 breast cancer cells by inhibiting STAT3 signaling. Their study further highlights the ability of quercetin to prevent or treat HER2-overexpressing breast cancer [68]. Quercetin has demonstrated broad-spectrum anticancer activity against various cancer types, including lung, oral, breast, ovarian, colon, and osteosarcoma [65]. The present study shows a stable RMSD trajectory of the AKT1-Quercetin complex, suggesting high conformational stability within the active site. RMSF analysis suggests that quercetin inactivates AKT1 by acting as a clamp that seals the mouth of the active site pocket through strong hydrogen bonds at ASN: A: 54, GLN: A: 79. It also forms a hydrophobic interaction with the residue LEU: A: 210 situated deep within the pocket, completely blocking the target protein’s flexibility.Among the key phytochemicals of Vernolac, nigellidine is an indazole-type alkaloid [69]. Although experimental validation studying the mechanisms of action of the pure compound, particularly in cancer treatment, is currently limited, an *in silico* study highlights the highest binding effectiveness between nigellidine and HER2 [70]. The results of the present study revealed a strong binding affinity towards AKT1, a serine/threonine kinase that plays a pivotal role in cell proliferation, survival, and apoptosis. To the best of our knowledge, this is the first study to reveal a direct interaction between nigellidine and AKT1, particularly in the context of cancer. The molecular docking results demonstrated a strong binding within the AKT1 active site, and molecular dynamics simulations further confirmed the stability of the protein-ligand complex. This novel finding suggests that nigellidine may exert its anticancer effects by modulating the PI3K/AKT/mTOR pathway. A previous molecular docking study reports that nigellidine binds strongly to HDAC1, indicating its potential to inhibit histone deacetylase activity [71]. HDAC1 is involved in maintaining the survival of CML cells [72]. Importantly, the CML pathway was identified as significantly enriched in our KEGG pathway analysis, and HDAC1 was identified as a potential target of nigellidine. In this study, high conformational stability is observed via RMSD analysis. Similar to Quercetin- AKT1 complex, RMSF results of Nigellidine- AKT1 demonstrate a rigid, low-fluctuation lockdown of the central activation loop residue LEU:A:210, while the outer gateway at TRP: A:80 forms strong hydrogen bonding immobilizing the AKT1 protein. Therefore, the results of the present study further support this mechanistic rationale for nigellidine-mediated epigenetic interferences in CML.

In the present study, network pharmacology integrated with molecular docking and dynamics simulations revealed several novel compound-target interactions relevant to cancer therapy. The present study reports for the first time a strong binding affinity of 4,8,12,16-tetramethylheptadecan-4-olide and tricin to AKT1, a key cancer-related target. Molecular dynamics analysis reveals a mean RMSD of 0.35 ± 0.05 nm, indicating high conformational stability and a steady trajectory throughout 100 ns. Similar to Quercetin and Nigellidine, this compound inactivates AKT1 by rigidly locking down the activation loop LEU:A:210 and simultaneously forming hydrophobic interactions at the outer pocket mouth residue TRP: A:80. Tricin-AKT1 interaction has been previously studied in the context of cardiovascular and neuropathology, including premature ventricular beats [73] and cerebral ischemia/reperfusion injury [74]. However, the relevance of the tricin-AKT1 interaction in oncogenic signaling has been newly identified in this study.

Previous studies have reported the anticancer activity of cytochalasin H in lung cancer cells, through modulation of targets such as Bcl-xL, Bcl-2, caspase 3, Bax, p53, and signaling pathways including PI3K/Akt/P70S6K and ERK1/2 [75,76]. However, our study is the first to report CDK4 as a novel molecular target of cytochalasin H, suggesting an additional mechanism by which it may exert antiproliferative effects. RMSD analysis shows a mean RMSD of 0.29 ± 0.07 nm, indicating high stability in conformational structure throughout a 100 ns period. Through RMSF analysis, it is revealed that it can rigidly lockdown the hinge region at ARG:A:186 and freeze the inner pocket area of the target protein. These findings expand the understanding of Vernolac’s mechanism of action while suggesting new avenues for targeting key cancer-related proteins.

A polyherbal decoction comprising *N. sativa* seeds, *H. indicus* roots, and *S. glabra* rhizomes in equal proportions has been traditionally utilized for many years by a family of Ayurvedic physicians in Sri Lanka to treat cancers, reflecting its long-standing ethnopharmacological application in cancer therapy [22]. According to the literature, this decoction offers significant protection against chemically induced liver cancer in rats without notable toxicity [23,24]. The decoction has been previously demonstrated to have cytotoxic effects against human hepatoma (HepG2) cells [22,77]. Moreover, the anticancer activity of this decoction has been reported to be mediated through multiple mechanisms, including protection against oxidative damage [19], anti-inflammatory activity [21], and upregulation and downregulation of antiapoptotic and proapoptotic genes [22].

Although chemo- and radiotherapy remain standard treatment modalities in cancer management, their effectiveness is often compromised due to drug resistance, tumor radio-resistance, and severe side effects. In this context, phytochemicals have emerged as potent chemo- and radiosensitizers [78]. According to the literature, phytochemicals such as thymoquinone, quercetin, and lupeol have demonstrated strong chemosensitizing and radiosensitizing effects by modulating key molecular targets, including STAT3, AKT1, TP53, HIF-1α, and PTGS2 [79–81]. These phytochemicals exhibit their chemosensitizing effects via various signaling pathways such as JAK/STAT, NF-kB, Wnt, PI3K/AKT, and EGFR [81]. Phytochemicals such as quercetin have been demonstrated to radiosensitize cancer cells through the expression of *p53* and *Bax*, downregulation of *Bcl-2*, and induction of endoplasmic reticulum stress [79].

Chemo and radiotherapy are also associated with various side effects, severely affecting the quality of life of cancer patients. Quercetin, apigenin, ferulic acid, vitamin E, luteolin, and thymoquinone have been reported to demonstrate potent chemoprotective and radioprotective properties [81–83]. They exert this protectivity via a broad range of mechanisms, including antioxidation, anti-inflammation, myeloprotection, antimutagenesis, and immunomodulation [78]. The present network pharmacology-based study also revealed PTGS2, BCL2, PI3K, IL-6, TNF-α, AKT1, and VEGF as key targets within pathways associated with chemoprotection and radioprotection, further supporting the therapeutic relevance of Vernolac’s phytochemicals in alleviating treatment-induced toxicity. Moreover, this dual benefit was further supported by the present *in vitro* cytotoxicity studies. Vernolac demonstrated significant cytotoxicity against breast cancer, colorectal adenocarcinoma, and testicular embryonal carcinoma cells with minimal toxicity in non-tumorigenic mammary epithelial cells. Furthermore, the aforementioned decoction has previously demonstrated protection against bleomycin-induced cytogenetic damage in human peripheral blood lymphocytes (PBLs), indicating significant radioprotective capacity [17]. This suggests a distinct clinical advantage due to its potential to protect normal cells from radiation-induced damage. Notably, Vernolac contains the same three medicinal plants along with two additional components, offering enhanced therapeutic effects.

These findings suggest that Vernolac may not only exert anticancer effects and sensitizes cancer cells to therapy but also improve the quality of life in patients by ameliorating cancer therapy-induced toxicity.

Although the present network pharmacology-based study provides valuable preliminary insights into the potential anticancer effects of Vernolac as a nutraceutical for cancer patients, several limitations need to be acknowledged. The predictions derived from *in silico* target identification, network construction, molecular docking, and dynamics simulations remain theoretical. Although these analyses suggest plausible mechanisms, such as modulation of PI3K/Akt, MAPK, and apoptotic signaling, as well as chemoradiosensitizing potential, chemoprotection, and radioprotection, these predictions require rigorous *in vitro* and *in vivo* validation to confirm their biological relevance. Importantly, the present study did not consider the *in vivo* metabolic transformation of the phytochemicals present in Vernolac, which may influence their biological activity and bioavailability. Incorporating metabolomic or ADME-focused *in silico* tools could improve the predictive accuracy of future analyses.

It is important to note that molecular docking and molecular dynamics simulations were performed using the parent phytochemical structures. Many phytochemicals, such as flavonoids and triterpenoids, undergo extensive biotransformation, including gut microbiota metabolism and hepatic first-pass effects such as glucuronidation and sulfation [84]. These processes may alter their physicochemical and pharmacophore properties. Despite this limitation, parent structures are widely used in early-stage structure-based drug design to provide initial insights into potential binding modes and target interactions.

Moreover, future experimental work should prioritize validating the novel target-ligand interactions identified in the present study through molecular docking and dynamics simulations. Target-specific quantitative PCR (qPCR) assays can be employed to confirm the modulation of key genes identified through network analyses. In addition, cellular thermal shift assays (CETSA) would enable direct assessment of target engagement *in vitro*, providing biophysical evidence for the novel interactions identified between Vernolac compounds with protein targets. These techniques, together with subsequent functional assays and *in vivo* studies, will be essential to verify the novel compound-target relationships and mechanistic pathways proposed in this work.

## Conclusion

The present study highlights the multi-component, multi-target, and multi-pathway mechanisms underlying Vernolac’s anticancer effects. Through integrated network pharmacology, molecular docking, and molecular dynamics simulations, the results suggest that phytochemical constituents of Vernolac may collectively modulate several key cancer-associated pathways, including apoptosis, cell proliferation, immune modulation, inflammation and oxidative stress.

Moreover, the network analysis of Vernolac suggests a possible role in modulating drug resistance and treatment response, providing a computational basis for its application as a potential adjunct to conventional cancer therapies. This study is based on predictive computational approaches, and overall findings provide a mechanistic hypothesis for Vernolac while establishing a foundational framework linking compound–target–pathway interactions for future experimental validation in preclinical and clinical settings.

## Supporting information

S1 Table Identified phytochemical constituents of Vernolac targeting core cancer-related proteins. (XLSX)

S2 Table Results of the CytoNCA analysis of 137 core target proteins. (XLSX)

S3 Table Results of the MCODE analysis of the core protein-protein interaction network. (XLSX)

S4 Table Protein-ligand interaction data of the molecular docking study. (DOCX)

S5 Table Phytochemicals identified via GC-MS analysis. (PDF)
