# Supplementary material for: A network pharmacology-based approach and molecular docking study to explore the therapeutic potential of a nutraceutical formula (Vernolac) in the treatment of cancer
Source: PLoS One. 2026 Jul 1;21(7):e0352420. doi: 10.1371/journal.pone.0352420 (PMC13322568; doi:10.1371/journal.pone.0352420)
Supplement: S5 Table — (PDF) [file pone.0352420.s005.pdf]

TEST REPORT

Report No: (7424)290-0434 Dec 31, 2024

Attn: Dr. Srimal

**Customer:**	Fadna Life Science (Pvt) Ltd
**Address :**	106/6B, Araliya Uyana, Depanama, Pannipitiya, Sri Lanka.
**Date of Sample Received:**	Oct 16, 2024
**Date of Testing Started:**	Oct 16, 2024
**Date of Testing Completed:**	Dec 31, 2024
**Sample Description:**	**Sample Received as:**<br>A sample contained in a sealed bottle<br><br>**Sample Identified by the Client as:**<br>Vernolac Herbal Extraction<br>Expiry Date: 2025.08.25<br>Manufacture Date: 2024.08.25<br>Batch No: V240825
**Sample Drawn By BVCPS:**	NO

Photo of the Submitted Sample

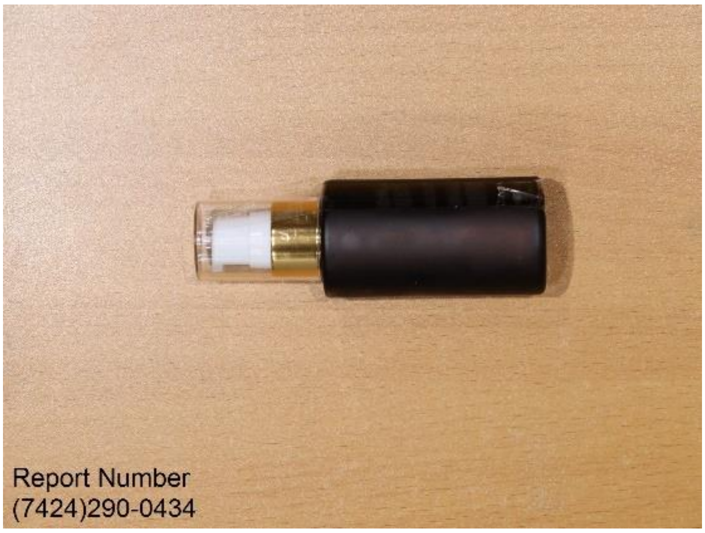

Bureau Veritas Consumer Products Services Lanka (Pvt) Ltd.

No. 570, Galle Road, Katubedda, Moratuwa, Sri Lanka
Tel: (9411) 2350111-115 (dedicated lines), Fax: (9411) 112622198 & 199
Email: bvcps.lanka@lk.bureauveritas.com

This report is governed by, and incorporates by reference, the Conditions of Testing as posted at the date of issuance of this report at http://www.bureauveritas.com/home/about-us/our-business/cps/about-us/terms-conditions/ and is intended for your exclusive use. Any copying or replication of this report to or for any other person or entity, or use of our name or trademark, is permitted only with our prior written permission. This report sets forth our findings solely with respect to the test samples identified herein. The results set forth in this report are not indicative or representative of the quality or characteristics of the lot from which a test sample was taken or any similar or identical product unless specifically and expressly noted. Our report includes all of the tests requested by you and the results thereof based upon the information that you provided to us. Measurement uncertainty is only provided upon request for accredited tests. Statements of conformity are based on simple acceptance criteria without taking measurement uncertainty into account, unless otherwise requested in writing. You have 60 days from date of issuance of this report to notify us of any material error or omission caused by our negligence or if you require measurement uncertainty; provided, however, that such notice shall be in writing and shall specifically address the issue you wish to raise. A failure to raise such issue within the prescribed time shall constitute you unqualified acceptance of the completeness of this report, the tests conducted and the correctness of the report contents.

The content of this PDF file is in accordance with the original issued reports for reference only.
This Test Report cannot be reproduced, except in full, without prior written permission of the company.

TEST REPORT

Report No: (7424)290-0434 Dec 31, 2024

TEST RESULTS

No.	Parameters	Results	Test Method
1	GCMS Analysis	Suspected compounds-<br>1. Alpha. -Tocopherol acetate<br>2. Alpha-pinene<br>3. Beta-Myrcene<br>4. Beta-pinene<br>5. D-Limonene<br>6. Dodecanoic acid<br>7. 1,6-Octadien-3-ol, 3,7-dimethyl-<br>8. Carvacrol<br>9. Cycloartenol<br>10. Thymoquinone<br>11. 1,3,5-Trimethyl benzene<br>12. Alpha terpinyl acetate<br>13. Hexadecanoic acid<br>14. Linalyl acetate<br>15. Stigmasterol<br>16. Octadecanoic acid<br>17. Oleic acid<br>18. Palmitic acid<br>19. Lauric acid<br>20. Acetic acid,phenylmethyl ester	In house method (GC-MS)

The content of this PDF file is in accordance with the original issued reports for reference only.
This Test Report cannot be reproduced, except in full, without prior written permission of the company.

TEST REPORT

Contact information for this report (Technical and General Inquiries and Feedback)

**GENERAL INQUIRIES:**		
ASHINI UDUGAMA	TEL: +94 740 385 038	E-MAIL: ashini.udugama@bureauveritas.com
**TECHNICAL INQUIRIES:**		
RUWANI AMARASINGHE	TEL: +94 768 229 457	E-MAIL: ruwani.amarasinghe@bureauveritas.com
**FEED BACK:**		
DALUKA DE SILVA– QUALITY ASSURANCE	TEL: +94 768 229 505	E-MAIL: daluka.desilva@bureauveritas.com

REVIEWED BY: DILINI JAYALATH

BUREAU VERITAS CONSUMER PRODUCTS SERVICES LANKA (PVT) LTD.
AUTHORIZED SIGNATORY

APPROVED BY: ______________________
RUWANI AMARASINGHE

DEPUTY MANAGER –
FOOD & MICROBIOLOGY LABORATORY

END OF THE REPORT.

The content of this PDF file is in accordance with the original issued reports for reference only.
This Test Report cannot be reproduced, except in full, without prior written permission of the company.
