# Supplementary material for: A network pharmacology-based approach and molecular docking study to explore the therapeutic potential of a nutraceutical formula (Vernolac) in the treatment of cancer
Source: PLoS One. 2026 Jul 1;21(7):e0352420. doi: 10.1371/journal.pone.0352420 (PMC13322568; doi:10.1371/journal.pone.0352420)
Supplement: Attachment — Submitted filename: Response to reviewers.docx [file pone.0352420.s008.docx]

**Response to reviewers**

We have revised the manuscript accordingly and provided detailed responses below.

| **No.** | **Comment** | **Response** |
| --- | --- | --- |
| **1** | The manuscript initially constructed a large virtual ligand library based on a database, and used this as the basis for high-throughput virtual screening (HTVS) and docking scoring (Table 5). However, in the subsequent GC-MS analysis (Table S5), some lead compounds with excellent docking affinity (such as Nigellidine) did not show high abundance, or were even not detected. It is recommended to add a discussion section on the "chemical spatial overlap between the virtual ligand library and the actual extracts". | We thank the reviewer for highlighting the discrepancy between the virtual screening results and the GC-MS profile. We would like to clarify that the GC-MS analysis in this study was intended to provide a **chemical fingerprint** of the extract rather than a comprehensive identification of all phytochemical constituents. As GC-MS primarily detects volatile and semi-volatile compounds, certain polar or non-volatile phytochemicals may not be captured.  In contrast, the virtual ligand library represents a broader theoretical phytochemical space compiled from literature and databases, independent of compound abundance. Therefore, compounds such as Nigellidine may exhibit high in silico binding affinity despite low detectability by GC-MS. We have now included a discussion in the revised manuscript to clarify this distinction and to emphasize that GC-MS and virtual screening provide complementary insights.  From line number 745 to 756. |
| **2** | When constructing the core PPI network, a degree centrality (DC) ≥ 20 was used as a hard filtering threshold. In network pharmacology, such a one-size-fits-all parameter setting, without underlying data support, is easily seen as introducing subjective bias. It is recommended to supplement the methodological or results section with the topological or statistical basis for this threshold setting. For example, the scale-free property of the initial network could be briefly explained, and based on the median, mean, or specific quantiles (such as the Top 20%) of the degree distribution of all nodes, it could be demonstrated how the DC ≥ 20 cutoff value can filter out edge noise (low connectivity nodes) while maximally preserving the core target group with biological information flow hub characteristics. | A justification for the use of the DC ≥ 20 threshold was already provided in the Methods section of the original manuscript. In the revised version, we have further strengthened this explanation by incorporating a more explicit, data-driven rationale based on the degree distribution of the network. Specifically, we clarified that a threshold representing approximately the top 20% of nodes was applied to identify highly connected targets, thereby reducing low-connectivity noise while preserving biologically relevant hub proteins. These additions have been included to improve transparency and address the concern regarding potential subjectivity in parameter selection.  From line number 175 to 182. |
| **3** | The analyses of 100 ns molecular dynamics trajectories are somewhat limited, only providing macroscopic reports on global RMSD and residue-level RMSF averages. They fail to fully utilize MD simulations to reveal target-ligand recognition mechanisms. By combining Figures 13 and 14, we can delve deeper into the local conformational fluctuations of one or two core complexes. Consideration could be given to supplementing these with microscopic explorations based on steric hindrance and hydrogen bond network occupancy. For example, do residues exhibiting significant rigidity (low fluctuation) in the RMSF map precisely map to catalytic triplet structures, allosteric sites, or key binding channels (such as activation loops)? How does ligand anchoring restrict conformational inversion in specific flexible regions? | We have expanded the MD simulation discussion beyond the global RMSD and RMSF analyses. Figures 13 and 14 are now jointly interpreted to better explain local conformational fluctuations and target–ligand recognition mechanisms. We also included a deeper discussion on residue rigidity in functionally important regions, ligand-induced stabilization of flexible domains, and the influence of ligand anchoring on conformational behavior within the binding pocket. These additions provide a more detailed mechanistic understanding of the stability and dynamic behavior of the protein–ligand complexes.  Results (from lines 626 to 746) and discussion sections. |
| **4** | The abstract and conclusion sections make overly absolute assertions regarding "chemoradiosensitizing" and "radioprotection." It's important to note that KEGG-based pathway enrichment analysis only provides statistically significant association hypotheses. Directly extrapolating clinical-level phenotypes without validation in cell or animal models of combined drug use (e.g., Vernolac + cisplatin/radiation intervention) is unsound. It is recommended to weaken such statements globally and revise them to phrases like "Based on the multi-pathway network topology, this study suggests that this combination therapy has the potential to intervene in drug resistance-related signaling axes," or "Its multi-target characteristics provide a computational theoretical hypothesis for subsequent exploration of adjuvant chemoprotective agents." | We have carefully revised the manuscript to address concerns regarding overly absolute statements in both the Introduction (lines 42–48) and Conclusion (lines 929–940). Specifically, we have softened the language to avoid overinterpretation of computational predictions and to ensure that conclusions remain appropriately aligned with the scope of the study.  In the Introduction, we revised the wording to present the study objectives in a more balanced and hypothesis-driven manner. In the Conclusion, we have rephrased the statements to emphasize that the findings are based on predictive computational approaches, while acknowledging that they provide a mechanistic hypothesis and a foundational framework for future experimental validation in preclinical and clinical settings. These revisions ensure that all interpretations remain consistent with the computational nature of the study and avoid overstating biological or clinical implications. |
| **5** | Although the study used SwissADME for preliminary filtering of oral bioavailability, there are inherent limitations to directly using "parent compounds" for target docking in structure-based drug design (SBDD) logic. Many phytochemicals (especially flavonoids and triterpenoids) undergo strong gut microbiota metabolism and first-pass effects in the liver (such as extensive glucuronidation or sulfation modifications) in vivo. It is recommended to proactively point out in the "Discussion" section that the molecular docking and MD simulation in this study were based on the ideal physical state of the prototype molecule, while the actual active metabolites entering the bloodstream may exhibit drastically different pharmacophore characteristics and steric hindrance. | We agree that in vivo metabolism may significantly alter phytochemical structures before systemic circulation. In response, we have added a clarification in the Discussion section highlighting that molecular docking and molecular dynamics simulations were performed using parent compounds under idealized conditions. We further acknowledge that extensive biotransformation (including gut microbiota metabolism and hepatic first-pass effects such as glucuronidation and sulfation) may generate metabolites with altered pharmacophore properties. This limitation has now been explicitly stated to ensure appropriate interpretation of the computational findings.  From line number 1009-1015 |
