# Supplementary material for: A network pharmacology-based approach and molecular docking study to explore the therapeutic potential of a nutraceutical formula (Vernolac) in the treatment of cancer
Source: PLoS One. 2026 Jul 1;21(7):e0352420. doi: 10.1371/journal.pone.0352420 (PMC13322568; doi:10.1371/journal.pone.0352420)
Supplement: Peer review history

# Peer review: Decision Letter 0

**DOI:** 10.1371/journal.pone.0352420.r001

**Authors:** Prakash Palaniswamy

3 Nov 2025

PONE-D-25-50180

A network pharmacology-based approach and molecular docking study to explore the therapeutic potential of a nutraceutical formula (Vernolac) in the treatment of cancer

PLOS ONE

Dear Dr. Samarakoon,

Thank you for submitting your manuscript to PLOS ONE. After careful consideration, we feel that it has merit but does not fully meet PLOS ONE’s publication criteria as it currently stands. Therefore, we invite you to submit a revised version of the manuscript that addresses the points raised during the review process.

Please submit your revised manuscript by Dec 18 2025 11:59PM. If you will need more time than this to complete your revisions, please reply to this message or contact the journal office at plosone@plos.org. When you're ready to submit your revision, log on to https://www.editorialmanager.com/pone/ and select the 'Submissions Needing Revision' folder to locate your manuscript file.

Please include the following items when submitting your revised manuscript:

- A rebuttal letter that responds to each point raised by the academic editor and reviewer(s). You should upload this letter as a separate file labeled 'Response to Reviewers'.
- A marked-up copy of your manuscript that highlights changes made to the original version. You should upload this as a separate file labeled 'Revised Manuscript with Track Changes'.
- An unmarked version of your revised paper without tracked changes. You should upload this as a separate file labeled 'Manuscript'.

If you would like to make changes to your financial disclosure, please include your updated statement in your cover letter. Guidelines for resubmitting your figure files are available below the reviewer comments at the end of this letter.

If applicable, we recommend that you deposit your laboratory protocols in protocols.io to enhance the reproducibility of your results. Protocols.io assigns your protocol its own identifier (DOI) so that it can be cited independently in the future. For instructions see: https://journals.plos.org/plosone/s/submission-guidelines#loc-laboratory-protocols. Additionally, PLOS ONE offers an option for publishing peer-reviewed Lab Protocol articles, which describe protocols hosted on protocols.io. Read more information on sharing protocols at https://plos.org/protocols?utm_medium=editorial-email&utm_source=authorletters&utm_campaign=protocols.

We look forward to receiving your revised manuscript.

Kind regards,

Prakash Palaniswamy, Ph.D

Academic Editor

PLOS ONE

Journal Requirements:

When submitting your revision, we need you to address these additional requirements.

1. Please ensure that your manuscript meets PLOS ONE's style requirements, including those for file naming. The PLOS ONE style templates can be found at

https://journals.plos.org/plosone/s/file?id=wjVg/PLOSOne_formatting_sample_main_body.pdf and https://journals.plos.org/plosone/s/file?id=ba62/PLOSOne_formatting_sample_title_authors_affiliations.pdf

2. Please include captions for your Supporting Information files at the end of your manuscript, and update any in-text citations to match accordingly. Please see our Supporting Information guidelines for more information: http://journals.plos.org/plosone/s/supporting-information

If the reviewer comments include a recommendation to cite specific previously published works, please review and evaluate these publications to determine whether they are relevant and should be cited. There is no requirement to cite these works unless the editor has indicated otherwise.

Additional Editor Comments:

Major Comments

1. Novelty and Rationale

o The rationale for selecting Vernolac as the focus of this study needs to be better justified. Please elaborate on the known phytochemical composition of Vernolac and its traditional or reported pharmacological relevance to cancer treatment.

o The introduction should clearly highlight the novelty of this work compared to previous network pharmacology and molecular docking studies of similar nutraceutical formulations.

2. Compound Identification

o The manuscript does not specify how the active compounds of Vernolac were identified (e.g., LC–MS, GC–MS, or literature-based databases such as TCMSP, PubChem, or ChemSpider). Please clarify the selection criteria and provide a detailed table listing compound names, molecular IDs, and ADMET properties.

o If compounds were retrieved from literature or databases only, experimental validation (e.g., phytochemical profiling) should be discussed as a limitation.

3. Network Pharmacology Workflow

o The workflow for target prediction and network construction lacks clarity. Please describe the databases used (e.g., SwissTargetPrediction, STRING, DisGeNET) and the parameters applied for filtering targets.

o The methods for PPI network construction, topological analysis, and hub gene identification (e.g., degree centrality, betweenness centrality) need to be explicitly described.

o The results of GO and KEGG pathway enrichment should be accompanied by p-values or FDR-adjusted significance levels.

4. Molecular Docking Methodology

o The docking protocol should include the PDB IDs of target proteins, docking software (e.g., AutoDock Vina, Glide), and grid box dimensions. Validation of docking accuracy (e.g., redocking RMSD values) should be reported.

o The binding affinity results should be compared with known standard or reference ligands to contextualize the docking scores.

5. Biological Interpretation

o The discussion section needs deeper biological interpretation. How do the predicted pathways (e.g., PI3K/Akt, MAPK, apoptosis) relate to cancer hallmarks and possible mechanisms of Vernolac action?

o The study lacks in vitro or in vivo validation. The authors should discuss this limitation and, if possible, suggest a roadmap for future experimental validation.

6. Figures and Data Presentation

o Network figures are not sufficiently legible. Consider enlarging node labels and differentiating compound–target–pathway relationships with clearer color codes.

o Include a schematic summary of the study workflow (compound selection → target prediction → network analysis → docking → interpretation).

Minor Comments

1. Please ensure consistent use of gene/protein names (e.g., use official HGNC gene symbols throughout).

2. Some references are outdated; include recent (2023–2025) studies on network pharmacology and nutraceuticals in oncology.

3. Check for grammatical and typographical errors throughout the manuscript.

4. The abstract should include quantitative results (e.g., number of compounds, hub genes identified, top docking scores).

5. Provide a clear statement of limitations and future directions in the conclusion.

Strengths:

The study uses an integrative in silico approach combining network pharmacology and molecular docking, which is appropriate for exploring multi-component nutraceuticals.

The findings could provide a foundation for experimental validation.

Weaknesses:

Methodological transparency and reproducibility need improvement.

Biological significance of results should be better connected to cancer mechanisms.

Figures and tables require clearer formatting.

Recommendation: Major Revision

[Note: HTML markup is below. Please do not edit.]

Reviewers' comments:

Reviewer's Responses to Questions

**Comments to the Author**

1. Is the manuscript technically sound, and do the data support the conclusions?

The manuscript must describe a technically sound piece of scientific research with data that supports the conclusions. Experiments must have been conducted rigorously, with appropriate controls, replication, and sample sizes. The conclusions must be drawn appropriately based on the data presented.

Reviewer #1: Partly

**********

2. Has the statistical analysis been performed appropriately and rigorously?

Reviewer #1: N/A

**********

3. Have the authors made all data underlying the findings in their manuscript fully available?

The PLOS Data policy requires authors to make all data underlying the findings described in their manuscript fully available without restriction, with rare exception (please refer to the Data Availability Statement in the manuscript PDF file). The data should be provided as part of the manuscript or its supporting information, or deposited to a public repository. For example, in addition to summary statistics, the data points behind means, medians and variance measures should be available. If there are restrictions on publicly sharing data—e.g. participant privacy or use of data from a third party—those must be specified.

Reviewer #1: Yes

**********

4. Is the manuscript presented in an intelligible fashion and written in standard English?

PLOS ONE does not copyedit accepted manuscripts, so the language in submitted articles must be clear, correct, and unambiguous. Any typographical or grammatical errors should be corrected at revision, so please note any specific errors here.

Reviewer #1: No

**********

5. Review Comments to the Author

Please use the space provided to explain your answers to the questions above. You may also include additional comments for the author, including concerns about dual publication, research ethics, or publication ethics. (Please upload your review as an attachment if it exceeds 20,000 characters)

Reviewer #1: Authors should rewrite the manuscript completely. The methods and results provided are quiet unclear. As the invitro study is presented with Vernolactone present the results and discussion in focus of that conclusion. Move others to the supplementary file section.

**********

6. PLOS authors have the option to publish the peer review history of their article (what does this mean?). If published, this will include your full peer review and any attached files.

If you choose “no”, your identity will remain anonymous but your review may still be made public.

**Do you want your identity to be public for this peer review?**  For information about this choice, including consent withdrawal, please see our Privacy Policy.

Reviewer #1: No

**********

[NOTE: If reviewer comments were submitted as an attachment file, they will be attached to this email and accessible via the submission site. Please log into your account, locate the manuscript record, and check for the action link "View Attachments". If this link does not appear, there are no attachment files.]

To ensure your figures meet our technical requirements, please review our figure guidelines: https://journals.plos.org/plosone/s/figures

You may also use PLOS’s free figure tool, NAAS, to help you prepare publication quality figures: https://journals.plos.org/plosone/s/figures#loc-tools-for-figure-preparation.

NAAS will assess whether your figures meet our technical requirements by comparing each figure against our figure specifications.

# Author comment: Author response to Decision Letter 1

**DOI:** 10.1371/journal.pone.0352420.r002

17 Dec 2025

Academic Editor's comments

Comment: Please ensure that your manuscript meets PLOS ONE's style requirements, including those for file naming.

Response: We have carefully reviewed and revised the manuscript to ensure full compliance with PLOS ONE’s style requirements, including file naming conventions.

Comment:Please include captions for your Supporting Information files at the end of your manuscript, and update any in-text citations to match accordingly. Please see our Supporting Information guidelines for more information

Response: The captions for all Supporting Information files have been added at the end of the manuscript, and all in-text citations have been updated accordingly in line with the Supporting Information guidelines.

Comment: The rationale for selecting Vernolac as the focus of this study needs to be better justified.

Response: The rationale for selecting Vernolac has now been substantially expanded in the Introduction (From line 69 to 105). Additional text clarifies its ethnopharmacological relevance, the documented anticancer activities of its constituent plants, its traditional use in Sri Lankan Traditional Ayurvedic practice, and its current use as a commercially available polyherbal nutraceutical for cancer management (from line 78 to 90). We have also highlighted the absence of mechanistic studies on Vernolac, establishing a clear justification for its selection (from line 91 to 105).

Comment: Please elaborate on the known phytochemical composition of Vernolac and its traditional or reported pharmacological relevance to cancer treatment.

Response: We have expanded the Introduction and Discussion sections to include detailed information on the known phytochemical constituents of the five plants present in Vernolac, supported by relevant literature. We have also elaborated on the documented anticancer activities and traditional medicinal relevance of these plants, thereby strengthening the contextual basis for the study findings (Introduction: from line 78 to 105) (Discussion section: from 717 to 876).

Comment:The introduction should clearly highlight the novelty of this work compared to previous network pharmacology and molecular docking studies of similar nutraceutical formulations.

Response: We agree with the reviewer and have revised the Introduction to articulate the novelty of this work clearly (from line 78 to 105). The updated text emphasizes that this study represents the first network pharmacology–based analysis of a Sri Lankan herbal formulation, distinguishing Vernolac from previously studied Indian and Chinese polyherbal formulations (from line 69 to 77). The unique ethnopharmacological background of Vernolac, combined with the absence of prior mechanistic investigations, is now highlighted to clearly present the novelty and significance of the study (from line 85 to 102).

The manuscript does not specify how the active compounds of Vernolac were identified (e.g., LC–MS, GC–MS, or literature-based databases such as TCMSP, PubChem, or ChemSpider). Please clarify the selection criteria and provide a detailed table listing compound names, molecular IDs, and ADMET properties.

Comment: If compounds were retrieved from literature or databases only, experimental validation (e.g., phytochemical profiling) should be discussed as a limitation.

Response: We have now included a comprehensive explanation of how the active compounds were identified in the Method section. Specifically, the phytochemical constituents of the five plants in Vernolac were retrieved from the IMPPAT database and literature (from line 112 to 122). In addition, GC–MS analysis was performed for experimental validation of the major constituents, and these results have already been incorporated into the Methods and Results sections for clarity (methods: from line 298 to 315; results: from line 693 to 697).

The list of major compounds identified by GC–MS analysis is now clearly presented in the Results section (Lines 693–697). Importantly, several of the major constituents detected experimentally, such as thymoquinone, carvacrol, and stigmasterol, were also included in the curated compound library used for network pharmacology analysis. These compounds emerged as key contributors in network construction, pathway enrichment, and molecular docking, where they showed strong interactions with cancer-relevant targets. This concordance between GC–MS profiling and in silico predictions strengthens the biological relevance of the network analysis and supports the proposed anticancer mechanisms of Vernolac. The complete list of GC-MS results is attached in S5 Table. The major compounds targeting the core 137 cancer-related targets, along with their PubChem IDs, molecular weight, and druglikeness, are mentioned in S1 Table.

Comment: The workflow for target prediction and network construction lacks clarity. Please describe the databases used (e.g., SwissTargetPrediction, STRING, DisGeNET) and the parameters applied for filtering targets.

Response: We appreciate the reviewer’s concern. The workflow for target prediction and network construction, including all databases and filtering parameters used, is described in detail in the Methods section. This includes the use of SwissTargetPrediction for compound–target identification, STRING for protein–protein interaction network construction, and GeneCards for cancer-related target retrieval, along with the specific confidence scores and selection thresholds applied (from line 137 to 186).

We have rechecked the section to ensure clarity and coherence, and the existing descriptions comprehensively cover the steps and parameters requested by the reviewer.

Comment: The methods for PPI network construction, topological analysis, and hub gene identification (e.g., degree centrality, betweenness centrality) need to be explicitly described.

Response: We thank the reviewer for highlighting the importance of methodological clarity. The manuscript provides an explicit, step-by-step description of the PPI network construction (from line 150 to 181), topological analyses (from line 182 to 186), hub-gene identification (from line 187 to 196), and clustering analysis (from line 197 to 208) in the Methods section. This text details the databases and confidence thresholds used to build the PPI, the network construction workflow, and the filtering steps applied to generate the final networks. It further specifies the topological metrics calculated (including degree centrality and betweenness centrality), the software used (Cytoscape) and the exact Cytoscape plugins employed for analysis, together with their URLs. We have reviewed that section for clarity and completeness to ensure it addresses the reviewer’s concern.

Comment: The results of GO and KEGG pathway enrichment should be accompanied by p-values or FDR-adjusted significance levels.

Response: We appreciate the reviewer’s suggestion. The GO and KEGG enrichment results in the manuscript already include the FDR values generated during the enrichment analysis. The full dataset is provided in the Results section (from line 466 to 494). We have rechecked the relevant sections to ensure that the reporting of FDR values is clear and consistently presented.

Comment: The docking protocol should include the PDB IDs of target proteins, docking software (e.g., AutoDock Vina, Glide), and grid box dimensions. Validation of docking accuracy (e.g., redocking RMSD values) should be reported.

Response: We appreciate the reviewer’s comment. The revised manuscript now clarifies all requested methodological details. The PDB IDs of all docked protein targets have been explicitly stated in the docking section (from line 238 to 245). Grid box dimensions have been added (from line 261 to 262), and the complete set of docking parameters and software specifications (including the tools used, their versions, and all applied settings) is described in detail in the Methods (from line 246 to 261). In addition, docking accuracy was evaluated through redocking (methods: from line 263 to 278), and the corresponding RMSD values are reported and discussed in the Results section (from line (from line 597 to 615). These updates ensure full transparency and reproducibility of the docking workflow.

Comment: The binding affinity results should be compared with known standard or reference ligands to contextualize the docking scores.

Response: Thank you for this observation. The manuscript includes a comparison of the docking scores of the Vernolac-derived compounds with the binding affinities of known standard/reference ligands for each target. These comparisons are presented in the Results section to contextualize the binding performance of the test compounds (results section: from line 519 to 590). We have reviewed the section again to ensure that these reference comparisons are clearly stated and easy to identify.

Comment: The discussion section needs deeper biological interpretation. How do the predicted pathways (e.g., PI3K/Akt, MAPK, apoptosis) relate to cancer hallmarks and possible mechanisms of Vernolac action?

Response: We thank the reviewer for this insightful comment. The Discussion section now provides an expanded biological interpretation of the predicted pathways, including how PI3K/Akt, MAPK, apoptosis regulation, and other enriched signaling routes align with established cancer hallmarks. We have discussed how these pathways may plausibly mediate the anticancer effects of Vernolac based on the identified bioactive compounds and their predicted targets. This interpretation connects the network findings with potential mechanisms of action and strengthens the translational relevance of the results (from line 736 to 876). We have also rechecked the section from the beginning to ensure these connections are clearly articulated.

Comment: The study lacks in vitro or in vivo validation. The authors should discuss this limitation and, if possible, suggest a roadmap for future experimental validation.

Response: We appreciate this important observation. The limitations regarding the absence of in vitro and in vivo validation have now been explicitly addressed and expanded in the revised manuscript. The end of the Discussion section includes a detailed limitations paragraph outlining the need for experimental confirmation of the predicted compound–target interactions, pathway involvement, and proposed mechanisms (from line 877 to 887). Additionally, we have added a clear roadmap for future work, highlighting the necessity of conducting targeted in vitro assays, in vivo studies, and metabolomic profiling to validate the computational predictions (from line 888 to 896). These points are further reinforced in the Conclusion, which now emphasizes the direction for subsequent experimental investigations (from line 897 to 907). Together, these revisions address the reviewer’s concern and clarify the study’s future trajectory.

Comment: Network figures are not sufficiently legible. Consider enlarging node labels and differentiating compound–target–pathway relationships with clearer color codes. Include a schematic summary of the study workflow (compound selection → target prediction → network analysis → docking → interpretation).

Response: We thank the reviewer for this valuable suggestion. The network figures in the manuscript have already been formatted for clarity, with enlarged node labels and distinct color coding to differentiate compound–target–pathway relationships. Additionally, a schematic workflow illustrating the full study pipeline—from compound selection and target prediction to network construction, enrichment analysis, docking, and final interpretation—was included in the manuscript (line 108-figure). We have reviewed all figures again to ensure that their presentation remains clear and visually accessible.

Comment: Please ensure consistent use of gene/protein names (e.g., use official HGNC gene symbols throughout).

Response: We have carefully reviewed the entire manuscript and corrected all gene and protein names to ensure consistent use of the official HGNC gene symbols throughout.

Comment: Some references are outdated; include recent (2023–2025) studies on network pharmacology and nutraceuticals in oncology.

Response: Thank you for the suggestion. Several recent studies published between 2023 and 2025 on network pharmacology and polyherbal formulations have been incorporated into the Introduction, Results, and Discussion sections to strengthen the scientific context (lines 74, 77, and 85).

Comment: Check for grammatical and typographical errors throughout the manuscript.

Response: The entire manuscript has been thoroughly revised for grammatical accuracy, clarity, and typographical consistency.

Comment: The abstract should include quantitative results (e.g., number of compounds, hub genes identified, top docking scores).

Response: Thank you for the suggestion. The abstract has been revised to include key quantitative results, including the number of compounds identified (lines 31 to 32), predicted targets, hub genes, and major pathways highlighted by the analysis (lines 33 to 47). Results of the SRB assay are also present in the abstract (Lines 47 to 50).

Reviewer #1 Comments

Comment: Is the manuscript technically sound, and do the data support the conclusions?

The manuscript must describe a technically sound piece of scientific research with data that supports the conclusions. Experiments must have been conducted rigorously, with appropriate controls, replication, and sample sizes. The conclusions must be drawn appropriately based on the data presented.

Reviewer #1: Partly

Response: In the revised manuscript, we have addressed all concerns related to technical rigor and data interpretation. The methodology has been clarified in detail, appropriate validation steps (including docking, redocking and RMSD analysis) have been incorporated, and the conclusions have been carefully aligned with the presented data. We believe that the revised version now represents a technically sound study, with conclusions fully supported by the results.

Comment: Is the manuscript presented in an intelligible fashion and written in standard English?

PLOS ONE does not copyedit accepted manuscripts, so the language in submitted articles must be clear, correct, and unambiguous. Any typographical or grammatical errors should be corrected at revision, so please note any specific errors here.

Reviewer #1: No

Response: The manuscript has been thoroughly revised to improve clarity, coherence, and overall readability. All sections were carefully edited for standard English usage, grammar, syntax, and consistency. Typographical and grammatical errors have been corrected throughout the manuscript to ensure that it is now clear, unambiguous, and presented in an intelligible manner suitable for publication.

Comment:Authors should rewrite the manuscript completely. The methods and results provided are quiet unclear. As the in vitro study is presented with Vernolactone present the results and discussion in focus of that conclusion. Move others to the supplementary file section.

Response: In response to the reviewer’s concerns, the manuscript has been thoroughly revised for clarity, grammar, and organization. The Methods and Results sections have been rewritten to clearly distinguish between extract-based in vitro experiments and compound-level in silico analyses, ensuring consistency between experimental design, data interpretation, and conclusions. Where appropriate, methodological details and supporting data have been streamlined or moved to the Supplementary Information to improve readability.

We would like to clarify a possible misunderstanding. Vernolactone is one of the major constituents present in the Vernolac extract; however, the in vitro antiproliferative activity reported in this study was performed using the Vernolac extract as a whole, not the isolated pure compound Vernolactone. The SRB assay was conducted using the commercially available Vernolac extract to experimentally validate the anticancer potential of the polyherbal nutraceutical.

# Peer review: Decision Letter 1

**DOI:** 10.1371/journal.pone.0352420.r003

**Authors:** Prakash Palaniswamy, Yuliang Zhang

20 Apr 2026

PONE-D-25-50180R1

A network pharmacology-based approach and molecular docking study to explore the therapeutic potential of a nutraceutical formula (Vernolac) in the treatment of cancer

PLOS One

Dear Dr. Samarakoon,

Thank you for submitting your manuscript to PLOS ONE. After careful consideration, we feel that it has merit but does not fully meet PLOS ONE’s publication criteria as it currently stands. Therefore, we invite you to submit a revised version of the manuscript that addresses the points raised during the review process.

Please submit your revised manuscript by Jun 04 2026 11:59PM. If you will need more time than this to complete your revisions, please reply to this message or contact the journal office at plosone@plos.org. When you're ready to submit your revision, log on to https://www.editorialmanager.com/pone/ and select the 'Submissions Needing Revision' folder to locate your manuscript file.

Please include the following items when submitting your revised manuscript:

- A letter that responds to each point raised by the academic editor and reviewer(s). You should upload this letter as a separate file labeled 'Response to Reviewers'.
- A marked-up copy of your manuscript that highlights changes made to the original version. You should upload this as a separate file labeled 'Revised Manuscript with Track Changes'.
- An unmarked version of your revised paper without tracked changes. You should upload this as a separate file labeled 'Manuscript'.

If you would like to make changes to your financial disclosure, please include your updated statement in your cover letter. Guidelines for resubmitting your figure files are available below the reviewer comments at the end of this letter.

If applicable, we recommend that you deposit your laboratory protocols in protocols.io to enhance the reproducibility of your results. Protocols.io assigns your protocol its own identifier (DOI) so that it can be cited independently in the future. For instructions see: https://journals.plos.org/plosone/s/submission-guidelines#loc-laboratory-protocols. Additionally, PLOS ONE offers an option for publishing peer-reviewed Lab Protocol articles, which describe protocols hosted on protocols.io. Read more information on sharing protocols at https://plos.org/protocols?utm_medium=editorial-email&utm_source=authorletters&utm_campaign=protocols.

As the corresponding author, your ORCID iD is verified in the submission system and will appear in the published article. PLOS supports the use of ORCID, and we encourage all coauthors to register for an ORCID iD and use it as well. Please encourage your coauthors to verify their ORCID iD within the submission system before final acceptance, as unverified ORCID iDs will not appear in the published article. *Only*  the individual author can complete the verification step; PLOS staff *cannot*  verify ORCID iDs on behalf of authors.

We look forward to receiving your revised manuscript.

Kind regards,

Yuliang Zhang, Ph.D.

Academic Editor

PLOS One

Journal Requirements:

If the reviewer comments include a recommendation to cite specific previously published works, please review and evaluate these publications to determine whether they are relevant and should be cited. There is no requirement to cite these works unless the editor has indicated otherwise.

[Note: HTML markup is below. Please do not edit.]

Reviewers' comments:

Reviewer's Responses to Questions

**Comments to the Author**

1. If the authors have adequately addressed your comments raised in a previous round of review and you feel that this manuscript is now acceptable for publication, you may indicate that here to bypass the “Comments to the Author” section, enter your conflict of interest statement in the “Confidential to Editor” section, and submit your "Accept" recommendation.

Reviewer #2: (No Response)

**********

2. Is the manuscript technically sound, and do the data support the conclusions?

The manuscript must describe a technically sound piece of scientific research with data that supports the conclusions. Experiments must have been conducted rigorously, with appropriate controls, replication, and sample sizes. The conclusions must be drawn appropriately based on the data presented.

Reviewer #2: Yes

**********

3. Has the statistical analysis been performed appropriately and rigorously?

Reviewer #2: Yes

**********

4. Have the authors made all data underlying the findings in their manuscript fully available?

The PLOS Data policy requires authors to make all data underlying the findings described in their manuscript fully available without restriction, with rare exception (please refer to the Data Availability Statement in the manuscript PDF file). The data should be provided as part of the manuscript or its supporting information, or deposited to a public repository. For example, in addition to summary statistics, the data points behind means, medians and variance measures should be available. If there are restrictions on publicly sharing data—e.g. participant privacy or use of data from a third party—those must be specified.

Reviewer #2: Yes

**********

5. Is the manuscript presented in an intelligible fashion and written in standard English?

PLOS ONE does not copyedit accepted manuscripts, so the language in submitted articles must be clear, correct, and unambiguous. Any typographical or grammatical errors should be corrected at revision, so please note any specific errors here.

Reviewer #2: Yes

**********

6. Review Comments to the Author

Please use the space provided to explain your answers to the questions above. You may also include additional comments for the author, including concerns about dual publication, research ethics, or publication ethics. (Please upload your review as an attachment if it exceeds 20,000 characters)

Reviewer #2: This manuscript integrates network pharmacology analysis, molecular docking, and molecular dynamics (MD) simulations to elucidate the potential multi-target pharmacological mechanisms of Vernolac. The overall computational workload is substantial, but some areas require improvement.

1. The manuscript initially constructed a large virtual ligand library based on a database, and used this as the basis for high-throughput virtual screening (HTVS) and docking scoring (Table 5). However, in the subsequent GC-MS analysis (Table S5), some lead compounds with excellent docking affinity (such as Nigellidine) did not show high abundance, or were even not detected. It is recommended to add a discussion section on the "chemical spatial overlap between the virtual ligand library and the actual extracts".

2. When constructing the core PPI network, a degree centrality (DC) ≥ 20 was used as a hard filtering threshold. In network pharmacology, such a one-size-fits-all parameter setting, without underlying data support, is easily seen as introducing subjective bias. It is recommended to supplement the methodological or results section with the topological or statistical basis for this threshold setting. For example, the scale-free property of the initial network could be briefly explained, and based on the median, mean, or specific quantiles (such as the Top 20%) of the degree distribution of all nodes, it could be demonstrated how the DC ≥ 20 cutoff value can filter out edge noise (low connectivity nodes) while maximally preserving the core target group with biological information flow hub characteristics.

3. The analyses of 100 ns molecular dynamics trajectories are somewhat limited, only providing macroscopic reports on global RMSD and residue-level RMSF averages. They fail to fully utilize MD simulations to reveal target-ligand recognition mechanisms. By combining Figures 13 and 14, we can delve deeper into the local conformational fluctuations of one or two core complexes. Consideration could be given to supplementing these with microscopic explorations based on steric hindrance and hydrogen bond network occupancy. For example, do residues exhibiting significant rigidity (low fluctuation) in the RMSF map precisely map to catalytic triplet structures, allosteric sites, or key binding channels (such as activation loops)? How does ligand anchoring restrict conformational inversion in specific flexible regions?

4. The abstract and conclusion sections make overly absolute assertions regarding "chemoradiosensitizing" and "radioprotection." It's important to note that KEGG-based pathway enrichment analysis only provides statistically significant association hypotheses. Directly extrapolating clinical-level phenotypes without validation in cell or animal models of combined drug use (e.g., Vernolac + cisplatin/radiation intervention) is unsound. It is recommended to weaken such statements globally and revise them to phrases like "Based on the multi-pathway network topology, this study suggests that this combination therapy has the potential to intervene in drug resistance-related signaling axes," or "Its multi-target characteristics provide a computational theoretical hypothesis for subsequent exploration of adjuvant chemoprotective agents."

5. Although the study used SwissADME for preliminary filtering of oral bioavailability, there are inherent limitations to directly using "parent compounds" for target docking in structure-based drug design (SBDD) logic. Many phytochemicals (especially flavonoids and triterpenoids) undergo strong gut microbiota metabolism and first-pass effects in the liver (such as extensive glucuronidation or sulfation modifications) in vivo. It is recommended to proactively point out in the "Discussion" section that the molecular docking and MD simulation in this study were based on the ideal physical state of the prototype molecule, while the actual active metabolites entering the bloodstream may exhibit drastically different pharmacophore characteristics and steric hindrance.

**********

7. PLOS authors have the option to publish the peer review history of their article (what does this mean?). If published, this will include your full peer review and any attached files.

If you choose “no”, your identity will remain anonymous but your review may still be made public.

**Do you want your identity to be public for this peer review?**  For information about this choice, including consent withdrawal, please see our Privacy Policy.

Reviewer #2: No

**********

[NOTE: If reviewer comments were submitted as an attachment file, they will be attached to this email and accessible via the submission site. Please log into your account, locate the manuscript record, and check for the action link "View Attachments". If this link does not appear, there are no attachment files.]

To ensure your figures meet our technical requirements, please review our figure guidelines: https://journals.plos.org/plosone/s/figures

You may also use PLOS’s free figure tool, NAAS, to help you prepare publication quality figures: https://journals.plos.org/plosone/s/figures#loc-tools-for-figure-preparation.

NAAS will assess whether your figures meet our technical requirements by comparing each figure against our figure specifications.

# Author comment: Author response to Decision Letter 2

**DOI:** 10.1371/journal.pone.0352420.r004

4 Jun 2026

Comment 1: The manuscript initially constructed a large virtual ligand library based on a database, and used this as the basis for high-throughput virtual screening (HTVS) and docking scoring (Table 5). However, in the subsequent GC-MS analysis (Table S5), some lead compounds with excellent docking affinity (such as Nigellidine) did not show high abundance, or were even not detected. It is recommended to add a discussion section on the "chemical spatial overlap between the virtual ligand library and the actual extracts".

Response: We thank the reviewer for highlighting the discrepancy between the virtual screening results and the GC-MS profile. We would like to clarify that the GC-MS analysis in this study was intended to provide a chemical fingerprint of the extract rather than a comprehensive identification of all phytochemical constituents. As GC-MS primarily detects volatile and semi-volatile compounds, certain polar or non-volatile phytochemicals may not be captured.

In contrast, the virtual ligand library represents a broader theoretical phytochemical space compiled from literature and databases, independent of compound abundance. Therefore, compounds such as Nigellidine may exhibit high in silico binding affinity despite low detectability by GC-MS. We have now included a discussion in the revised manuscript to clarify this distinction and to emphasize that GC-MS and virtual screening provide complementary insights.

From line number 745 to 756.

Comment 2: When constructing the core PPI network, a degree centrality (DC) ≥ 20 was used as a hard filtering threshold. In network pharmacology, such a one-size-fits-all parameter setting, without underlying data support, is easily seen as introducing subjective bias. It is recommended to supplement the methodological or results section with the topological or statistical basis for this threshold setting. For example, the scale-free property of the initial network could be briefly explained, and based on the median, mean, or specific quantiles (such as the Top 20%) of the degree distribution of all nodes, it could be demonstrated how the DC ≥ 20 cutoff value can filter out edge noise (low connectivity nodes) while maximally preserving the core target group with biological information flow hub characteristics.

Response: A justification for the use of the DC ≥ 20 threshold was already provided in the Methods section of the original manuscript. In the revised version, we have further strengthened this explanation by incorporating a more explicit, data-driven rationale based on the degree distribution of the network. Specifically, we clarified that a threshold representing approximately the top 20% of nodes was applied to identify highly connected targets, thereby reducing low-connectivity noise while preserving biologically relevant hub proteins. These additions have been included to improve transparency and address the concern regarding potential subjectivity in parameter selection.

From line number 175 to 182.

Comment 3: The analyses of 100 ns molecular dynamics trajectories are somewhat limited, only providing macroscopic reports on global RMSD and residue-level RMSF averages. They fail to fully utilize MD simulations to reveal target-ligand recognition mechanisms. By combining Figures 13 and 14, we can delve deeper into the local conformational fluctuations of one or two core complexes. Consideration could be given to supplementing these with microscopic explorations based on steric hindrance and hydrogen bond network occupancy. For example, do residues exhibiting significant rigidity (low fluctuation) in the RMSF map precisely map to catalytic triplet structures, allosteric sites, or key binding channels (such as activation loops)? How does ligand anchoring restrict conformational inversion in specific flexible regions?

Response: We have expanded the MD simulation discussion beyond the global RMSD and RMSF analyses. Figures 13 and 14 are now jointly interpreted to better explain local conformational fluctuations and target–ligand recognition mechanisms. We also included a deeper discussion on residue rigidity in functionally important regions, ligand-induced stabilization of flexible domains, and the influence of ligand anchoring on conformational behavior within the binding pocket. These additions provide a more detailed mechanistic understanding of the stability and dynamic behavior of the protein–ligand complexes.

Results (from lines 626 to 746) and discussion sections.

Comment 4: The abstract and conclusion sections make overly absolute assertions regarding "chemoradiosensitizing" and "radioprotection." It's important to note that KEGG-based pathway enrichment analysis only provides statistically significant association hypotheses. Directly extrapolating clinical-level phenotypes without validation in cell or animal models of combined drug use (e.g., Vernolac + cisplatin/radiation intervention) is unsound. It is recommended to weaken such statements globally and revise them to phrases like "Based on the multi-pathway network topology, this study suggests that this combination therapy has the potential to intervene in drug resistance-related signaling axes," or "Its multi-target characteristics provide a computational theoretical hypothesis for subsequent exploration of adjuvant chemoprotective agents."

Response: We have carefully revised the manuscript to address concerns regarding overly absolute statements in both the Introduction (lines 42–48) and Conclusion (lines 929–940). Specifically, we have softened the language to avoid overinterpretation of computational predictions and to ensure that conclusions remain appropriately aligned with the scope of the study.

In the Introduction, we revised the wording to present the study objectives in a more balanced and hypothesis-driven manner. In the Conclusion, we have rephrased the statements to emphasize that the findings are based on predictive computational approaches, while acknowledging that they provide a mechanistic hypothesis and a foundational framework for future experimental validation in preclinical and clinical settings. These revisions ensure that all interpretations remain consistent with the computational nature of the study and avoid overstating biological or clinical implications.

Comment 5: Although the study used SwissADME for preliminary filtering of oral bioavailability, there are inherent limitations to directly using "parent compounds" for target docking in structure-based drug design (SBDD) logic. Many phytochemicals (especially flavonoids and triterpenoids) undergo strong gut microbiota metabolism and first-pass effects in the liver (such as extensive glucuronidation or sulfation modifications) in vivo. It is recommended to proactively point out in the "Discussion" section that the molecular docking and MD simulation in this study were based on the ideal physical state of the prototype molecule, while the actual active metabolites entering the bloodstream may exhibit drastically different pharmacophore characteristics and steric hindrance.

Response: We agree that in vivo metabolism may significantly alter phytochemical structures before systemic circulation. In response, we have added a clarification in the Discussion section highlighting that molecular docking and molecular dynamics simulations were performed using parent compounds under idealized conditions. We further acknowledge that extensive biotransformation (including gut microbiota metabolism and hepatic first-pass effects such as glucuronidation and sulfation) may generate metabolites with altered pharmacophore properties. This limitation has now been explicitly stated to ensure appropriate interpretation of the computational findings.

From line number 1009-1015

Attachment Submitted filename: Response to reviewers.docx

# Editor report: Decision Letter 2

**DOI:** 10.1371/journal.pone.0352420.r005

**Authors:** Prakash Palaniswamy, Yuliang Zhang, Yuliang Zhang

10 Jun 2026

A network pharmacology-based approach and molecular docking study to explore the therapeutic potential of a nutraceutical formula (Vernolac) in the treatment of cancer

PONE-D-25-50180R2

Dear Dr. Samarakoon,

We’re pleased to inform you that your manuscript has been judged scientifically suitable for publication and will be formally accepted for publication once it meets all outstanding technical requirements.

Within one week, you’ll receive an e-mail detailing the required amendments. When these have been addressed, you’ll receive a formal acceptance letter and your manuscript will be scheduled for publication.

An invoice will be generated when your article is formally accepted. Please note, if your institution has a publishing partnership with PLOS and your article meets the relevant criteria, all or part of your publication costs will be covered. Please make sure your user information is up-to-date by logging into Editorial Manager at Editorial Manager® and clicking the ‘Update My Information' link at the top of the page. For questions related to billing, please contact billing support.

If your institution or institutions have a press office, please notify them about your upcoming paper to help maximize its impact. If they’ll be preparing press materials, please inform our press team as soon as possible -- no later than 48 hours after receiving the formal acceptance. Your manuscript will remain under strict press embargo until 2 pm Eastern Time on the date of publication. For more information, please contact onepress@plos.org.

Kind regards,

Yuliang Zhang, Ph.D.

Academic Editor

PLOS One

Additional Editor Comments (optional):

Reviewers' comments:

# Editor report: Acceptance letter

**DOI:** 10.1371/journal.pone.0352420.r006

**Authors:** Prakash Palaniswamy, Yuliang Zhang, Yuliang Zhang

PONE-D-25-50180R2

PLOS One

Dear Dr. Samarakoon,

I'm pleased to inform you that your manuscript has been deemed suitable for publication in PLOS One. Congratulations! Your manuscript is now being handed over to our production team.

At this stage, our production department will prepare your paper for publication. This includes ensuring the following:

* All references, tables, and figures are properly cited

* All relevant supporting information is included in the manuscript submission,

* There are no issues that prevent the paper from being properly typeset

You will receive further instructions from the production team, including instructions on how to review your proof when it is ready. Please keep in mind that we are working through a large volume of accepted articles, so please give us a few days to review your paper and let you know the next and final steps.

Lastly, if your institution or institutions have a press office, please let them know about your upcoming paper now to help maximize its impact. If they'll be preparing press materials, please inform our press team within the next 48 hours. Your manuscript will remain under strict press embargo until 2 pm Eastern Time on the date of publication. For more information, please contact onepress@plos.org.

You will receive an invoice from PLOS for your publication fee after your manuscript has reached the completed accept phase. If you receive an email requesting payment before acceptance or for any other service, this may be a phishing scheme. Learn how to identify phishing emails and protect your accounts at https://explore.plos.org/phishing.

If we can help with anything else, please email us at customercare@plos.org.

Thank you for submitting your work to PLOS ONE and supporting open access.

Kind regards,

PLOS ONE Editorial Office Staff

on behalf of

Dr. Yuliang Zhang

Academic Editor

PLOS One
